# Organelle genome assembly, annotation, and comparative analyses of *Typha latifolia and T. domingensis:* two keystone species for wetlands worldwide

**DOI:** 10.3389/fpls.2024.1484531

**Published:** 2024-12-05

**Authors:** Thida Soe, Jiali Kong, Liyun Nie, Jie Wang, Dan Peng, Luke R. Tembrock, Zhiqiang Wu

**Affiliations:** ^1^ Shenzhen Branch, Guangdong Laboratory of Lingnan Modern Agriculture, Key Laboratory of Synthetic Biology, Ministry of Agriculture and Rural Affairs, Agricultural Genomics Institute at Shenzhen, Chinese Academy of Agricultural Sciences, Shenzhen, China; ^2^ State Key Laboratory of Crop Stress Adaptation and Improvement, School of Life Sciences, Henan University, Kaifeng, China; ^3^ School of Medical, Molecular and Forensic Sciences, Murdoch University, Perth, WA, Australia; ^4^ College of Agriculture, Center for Genomics and Biotechnology, Fujian Agriculture and Forestry University, Fuzhou, China; ^5^ Department of Agricultural Biology, Colorado State University, Fort Collins, CO, United States

**Keywords:** organellar genomes, phylogeny, comparative analysis, *Typha*, synteny

## Abstract

*Typha* is a cosmopolitan aquatic plant genus that includes species with widespread global distributions. In previous studies, a revised molecular phylogeny was inferred using seven plastid loci from nine *Typha* species across different geographic regions. By utilizing complete organellar genomes, we aim to provide a more comprehensive dataset that offers a robust phylogenetic signal for resolving *Typha* species evolutionary relationships. Here, we assembled *T. latifolia* and *T. domingensis* mitochondrial genomes (mitogenomes) using a combination of short-read and long-read data (PacBio, ONT). The mitogenomes of both species are assembled into single circular molecules of 395,136 bp and 395,140 bp in length, respectively, with a similar GC content of 46.7%. A total of 39 protein-coding genes, 17 tRNA genes, and 3 rRNA genes were annotated in both mitogenomes. The plastid genomes (plastomes) of both species possess typical quadripartite structures observed across most plants, with sizes of 161,545 bp and 161,230 bp. The overall average GC content of the plastomes of both species was 36.6%. The comparative analysis of the plastome and mitogenome revealed that 12 mitogenome DNA fragments share similar sequences with in the repeat regions of the corresponding plastomes, suggesting a past transfer of repeat regions into the mitogenome. Additionally, the mitogenomes of the two *Typha* species exhibited high sequence conservation with several syntenic blocks. Phylogenetic analysis of the organellar genomes of the two *Typha* species and 10 related species produced congruent phylogenetic trees. The availability of these organellar genomes from two *Typha* species provide valuable genetic resources for studying the evolution of Typhaceae and will improve taxonomic classifications within the family.

## Introduction

*Typha* (Typhaceae) is a perennial, monocot plant distributed worldwide in wetlands, lakes, and rivers (McNaughton, 1966). The APG II places Typhaceae in the order Poales within the Commelinid clade of the monocots (Ii, 2003). The family was previously consisted of a single genus (*Typha*). However, APG III included another genus, *Sparganium*, to this family (Angiosperm Phylogeny, 2009). The two genera have a total of 51 recognized species (Christenhusz and Byng, 2016). *Typha latifolia* (broadleaf cattail) and *Typha domingensis* (southern cattail) are prominent species within the genus *Typha*. *T. latifolia* has wide leaves and dense flower spikes, thriving in freshwater habitats, where it plays a keystone role by supporting wildlife and aiding nutrient cycling (Pandey and Verma, 2018). It is widely introduced for habitat restoration and soil stabilization due to its rhizome network (Grace and Harrison, 1986; Tornberg et al., 1994). In contrast, *T. domingensis*, with narrower leaves and fragmented flower spikes, inhabits tropical and subtropical regions and tolerates salinity, making it vital in coastal wetlands (Vivian et al., 2014; Bansal et al., 2019). *Typha* comprises 38 species, most of which have a widespread global distribution. (https://powo.science.kew.org/taxon/urn:lsid:ipni.org:names:30003823-2). However, taxonomic studies of *Typha* have often been confined to a specific regions, such as India (Saha, 1968), Europe (Volkova and Bobrov, 2022), Iran, Pakistan, Australia (Bokhari, 1983), North America (Kuehn and White, 1999), and China. The taxonomy of *Typha* has long been debated due to significant morphological variation and frequent interspecific hybridization (Smith, 1987). Since 1987, fifteen new species have been described, through all were based on local populations and lacked molecular data. A number of recent studies have provided complete plastome assemblies, and molecular phylogenies for the genus *Typha* (Liu et al., 2020). Up to now, only *T. latifolia* and *T. angustifolia* nuclear genomes were published in the genus *Typha* (Widanagama et al., 2022; Liao et al., 2023), and no more complete *Typha* genus mito genomes are available to date. Exploring the organelle genomes of *T. latifolia* and *T. domingensis* will help in the classification of *Typha* species and provide a genetic resource for further study of nucleocytoplasmic interaction.

Organellar genomes play a crucial role in an organism’s growth and development (Mahapatra et al., 2021). Plant organellar genomes employ high fidelity molecular mechanism to repair DNA damage and maintain of nucleotide sequences integrity, enabling them to withstand genotoxic stresses more effectively than nuclear genomes resulting in lower mutation rates (Maréchal and Brisson, 2010). Extensive analysis of organellar genomes has provided insights into the classification and evolution of various plant taxa (Wolfsberg et al., 2001). By 2023, the NCBI GenBank database contained approximately 13,000 plastomes but only 673 complete plant mitogenomes, with only 285 species having both organellar genomes sequenced (Wang et al., 2024b). The lower number of sequenced mitogenomes compared to plastomes is due to high repeat sequences, recombination, rearrangement rate, and the insertion of plastome sequences into the mitogenome (Johnston, 2019). Given these characteristics, the mitogenome is an effective model for analyzing genomic structure and evolutionary processes (Drouin et al., 2008). A previous molecular phylogenetic study outlined the relationships within *Typha* using sequences from seven plastid loci (Zhou et al., 2018). However, including additional taxa and genetic markers could enhance the resolution and support of subsequent phylogenetic analyses. Moreover, no studies have described a mitogenome from *Typha* or explored the exchange of DNA between the plastome and the mitogenome.

In this study, we assembled two *Typha* species (*T. latifolia* and *T. domingensis*) mitogenomes using previously published short-read and long-read (PacBio, ONT) data. We analyzed genomic characteristics, including gene content, repeat detection, codon usage, intracellular sequence transfer, mitogenomic synteny, and organellar phylogeny, to explore intrageneric variation in the maternally inherited organelles. The assembly of complete organellar genomes for these globally important species provides a foundational resource for further detailed studies of taxonomy, metabolic function, evolution, and molecular ecology.

## Materials and methods

### Data source

All raw sequence resources of both short and long-read from chloroplast and mitochondria were obtained from NCBI (https://www.ncbi.nlm.nih.gov/sra/). The raw sequencing data generated and analyzed during the current study of two *Typha* species were from BioProject PRJNA751759 and PRJNA742003. The *T. latifolia* plant was collected in Ontario, Canada, and *T. domingenis* plant was collected in the United States. Mitogenome and plastome sequences obtained from NCBI website were used for phylogenetic inference of the genus *Typha*, including the following species: *Spirodela polyrhiza* (NC_015891.1, NC_017840.1), *Butomus umbellatus* (NC_051949.1, NC_021399.1), *Zea mays* NB (NC_001666.2, AY506529.1), *Allium cepa* (OR783242.1, NC_030100.1), *Crocus sativus* (NC_041460.1, OL804177.1), *Zea perennis* (NC_030300.1, NC_008331.1), *Phoenix dactylifera* (NC_013991.2, NC_016740.1), *Oryza sativa Japonica* (LC739565.1, BA000029.3), *Oryza minuta* (MT726938.1, NC_029816.1), and *Arabidopsis thaliana* (AP000423, JF729202.1).

### Organelle genome assembly and annotation

The clean Illumina PE reads from whole-genome sequencing were first randomly selected using SeqKit 0.13.1 (Shen et al., 2016) to create datasets for mitogenome and plastome assembly. A combined strategy was performed to obtain accurate mitogenomes. The obtained Illumina reads were *de novo* assembled with using five distinct procedures in SPAdes 3.15.2 (Bankevich et al., 2012), wherein K-mer was then adjusted at five different values (51, 71, 91, 101, and 121) and obtained the assembled scaffolds. The coding sequence (CDS) of the *Oryza sativa* mitogenome (BA000029.3) was utilized as the query sequences to search against the draft scaffolds that were, visualized in Bandage 0.8.1 (Wick et al., 2015) to exclude non-mitogenomic scaffolds. Mitogenomic sequences from Illumina reads were eventually acquired after removing the fragments with abnormal depths as compared to general mitochondrial sequences (10 and more times lower, from the nuclear genome and 100 times and higher from the plastome). The mitochondrial sequences were used to select PacBio/ONT reads with the use of BLAST 2.11.0+ (Ye et al., 2006) with 80% identity, before which PacBio/ONT reads were self-corrected with the use of Nextdenovo 2.3.1 (https://github.com/Nextomics/NextDenovo). The corrected PacBio/ONT reads were *de novo* assembled using Flye 2.8.3 (Kolmogorov et al., 2019). The graph-based mitogenome was visualized by Bandage v0.8.1. For plastome assembly, SPAdes 3.15.2 was utilized for *de novo* assembly with five k-mer levels, as above. Bandage software (v.0.8.1) was employed to visualize the connections among contigs. Accurate annotations for the four organellar genomes were obtained using Geseq (https://chlorobox.mpimp-golm.mpg.de/geseq.html) with *Oryza sativa* (BA000029.3) and *Typha latifolia* (NC_013823.1) as references for the mitogenome and plastome, respectively. All annotations underwent manual verification and correction. The genome map was generated using OGDRAW1.3.1, (https://chlorobox.mpimpgolm.mpg.de/OGDraw.html).

### Repeats detection and codon usage analysis

Dispersed repeats were identified using the REPuter online program (https://bibiserv.cebitec.uni-bielefeld.de/reputer) with the following parameters: hamming distance = three, minimal repeat size = 30 (90% sequence identity or greater), maximum computed repeats = 5,000, and an e-value cutoff = 1e-5 (Kurtz et al., 2001). Simple sequence repeats (SSRs) were detected using MISA (Beier et al., 2017), incorporating motif sizes ranging from one to six nucleotide units, with repeat lower thresholds set to eight, five, four, three, three, and three repeat units for mono-, di-, tri-, tetra-, penta-, and hexa-nucleotide SSRs, respectively. Additionally, we used the default parameter for Max. Length of sequence between two SSRs to register as compound SSR, set to 100 base pairs, allowing accurate identification of compound SSRs within our data. REPuter was utilized to identify long repeats across the chloroplast genome with default settings. The relative synonymous codon usage (RSCU) of the mitogenome was computed using DAMBE 5.2.73 (Xia and Xie, 2001).

### Synteny and transfer fragment analysis

In order to identify transferred fragments between the plastome and mitogenome, BLAST 2.11.0+ was employed to search for homologous fragments with an e-value of 1e-5 and 80% identity cutoff, as described previously (Hong et al., 2021). Interspecies homologous regions were examined to demonstrate mitogenomic synteny between the two *Typha* species, with fragments shorter than 100 bp excluded from the analysis. The results were visualized using the Circos package within TBtools (Zhang et al., 2013).

### Organellar phylogenetical inference

Both the plastome and the mitogenome of two *Typha* species and 10 related species were subjected to phylogenetic analysis. Considering all the available mitogenomic data in NCBI, the mitogenome and plastome of 10 species that represent various clades or branches along the tree of life, including *Spirodela polyrhiza*, *Butomus umbellatus*, *Zea mays* NB, *Allium cepa*, *Crocus sativus*, *Zea perennis*, *Phoenix dactylifera*, *Oryza sativa Japonica*, *Oryza minuta* (MT726938.1, NC_029816.1), and *Arabidopsis thaliana* as the outgroup. All shared coding sequences (CDS) were aligned in MEGA 7 (Kumar et al., 2016). The aligned CDSs, consisting of 24 for the mitogenome and 77 for the plastome, were concatenated using Geneious Prime (https://www.geneious.com). Gblocks v.0.91b was utilized to select the optimal multiple sequence alignment regions with default parameters (Castresana, 2000). A Maximum Likelihood (ML) phylogenetic inference was conducted in IQ-Tree 2.1 (Nguyen et al., 2015) using the best evolutionary models, ‘TVM+F+I+G4’ for plastomes and ‘GTR+F+G4’ for mitogenomes, selected based on Bayesian Information Criterion scores. Clade support was assessed with 1,000 bootstrap (BS) replicates. The newick format tree was visualized using iTOL6 (https://itol.embl.de/).

## Results

### General features of the organellar genomes from two *Typha* species

**Figure 1:**
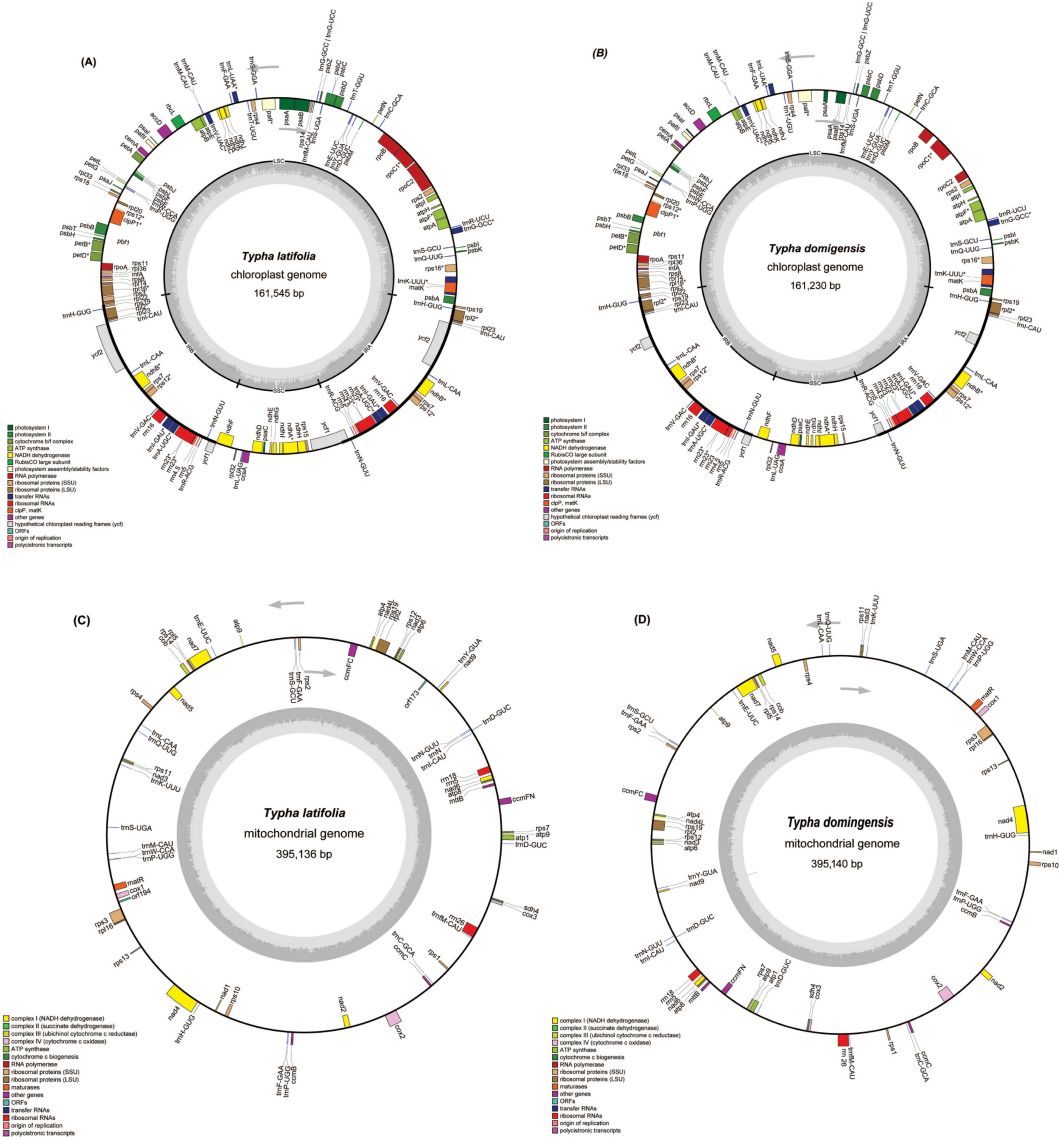
The circular maps of the organelle genomes of two *Typha* species. **(A)** The circular map of the plastome in *T. latifolia*. **(B)** The circular map of the plastome in *T. domingensis*. **(C)** The circular map of the mitogenome in *T. latifolia*. **(D)** The circular map of the mitogenome in *T. domingensis*. Genomic features transcribed clockwise and counter-clockwise are drawn on the inside and outside of the circle, respectively. Genes are color-coded based on their functional groups. GC content is represented on the inner circle by the dark gray plot.

To assemble the organellar genomes of *T. latifolia* and *T. domingensis*, we employed a combined approach using 1 Gb of Illumina reads, 86 Gb of PacBio reads and 20 Gb of ONT reads per sample. The results showed that the plastomes of *T. latifolia* and *T. domingensis* have the typical quadripartite structures like most flowering plants, with genome sizes of 161,545 bp and 161,230 bp respectively (**Figures 1A, B**). Both the plastomes were comprised of four distinctive parts in which the large single copy (LSC-, 89,131 bp, 88,964 bp) and the small single copy (SSC-, 18,554 bp, 18,503 bp) regions were separated by two inverted repeats (IRs, 26,930 bp, 26,881 bp). The overall average (GC) content of the plastomes was (36.6%) in both species. We discovered a total of 150 distinct genes in *T. latifolia*, consisting of 96 protein-coding genes, 10 rRNA genes, and 44 tRNA genes. In the assembled plastome of *T. domingensis*, we detected 141 distinct genes, with 89 protein-coding genes, 8 rRNA genes, and 44 tRNA genes (**Supplementary Tables S1**, **S2**). Seventeen genes contained one intron (*rpl2, ndhA, ndhB, rps12, rps16, rpl16, rrn23, rpoC1, petB, petD, atpF, trnG-GCC, trnL-UAA, trnV-UAC, trnA-UGC, trnI-GAU, trnK-UUU), and* two genes (*ycf3, clpP*) had two introns. The gene copy number was largely conserved between the two species, although *T. latifolia* exhibited higher copy numbers for several genes such as *trnI-GAU*, *rrn23S*, whereas *T. domingensis* showed increased copy numbers for *trnI-GAU* and *rps12*. We compared the plastomes using a dot plot. The results showed that the two plastomes were highly collinear indicating a conserved genomic architecture (**Supplementary Figure S1**). In both *T. latifolia* and *T. domingensis* mitogenomes, a single circular molecule was resolved, with sizes of 395,136 bp and 395,140 bp, respectively, and a GC content of 46.7% for both (**Figures 1C, D**). The nucleotide composition of the whole mitogenomes showed only slight variations, A, 27.0%; T, 26.04%; C, 23.03%; and G, 23.04% in *T. latifolia* and A, 26.04%; T, 27.0%; C, 23.04%; and G, 23.03% in *T. domingensis*. A total of 59 unique mitochondrial genes were located in *T. latifolia* and *T. domingensis*, and the two species shared 59 genes including 39 protein-coding genes (PCGs), three rRNA genes, and 17 tRNA genes. There were eleven genes that contained introns, with the details shown in **Table 1**. Among the eleven genes containing introns, the distribution is as follows: five genes possess one intron each (*ccmFC, rpl2, rps10, rps3, trnL-CAA*), one gene contains two introns (*cox2*), and one gene contains three introns (*nad4*). Additionally, four genes exhibit four introns each (*nad1, nad2, nad5, nad7*) in both *T. latifolia* and *T. domingensis* mitogenome.

**Table 1:** Known functional genes in the two *Typha* mitogenomes.

Gene functional categories	Genes names
ATP synthase	*atp1, atp4, atp6, atp8, atp9(×2)*
Cytochrome c biogenesis	ccmB, ccmC, ccmFC*, ccmFN
Apocytochrome b	*cob*
Cytochrome c oxidase	cox1, cox2**, cox3
Maturases	*matR*
Transport membrane protein	*mttB*
NADH dehydrogenase	nad1****, nad2****, nad3(×2), nad4***,
Large subunit of ribosomal proteins	nad4L, nad5****, nad6, nad7****, nad9, rpl16, rpl2*, rpl5
Small subunit of ribosomal proteins	rps1, rps10*, rps11, rps12, rps13, rps14, rps19, rps2, rps3*, rps4, rps7
Subunit of succinate dehydrogenase	*sdh4*
Ribosomal RNAs	*rrn18, rrn26, rrn5*
Transfer RNAs	*trnD-GUC* (×2), trnL-CAA*, trnP-UGG (×2)*, trnC-GCA, trnE-UUC, trnF-GAA* (×2)*, trnH-GUG, trnK-UUU, trnM-CAU, trnfM-CAU, trnI-CAU, trnN-GUU, trnQ-UUG, trnS-GCU, trnS-UGA, trnW-CCA, trnY-GUA*

* one intron, ** two introns, *** three introns, **** four introns; Gene (2): Number of copies of multi-copy genes.

### Repeat analysis

**Figure 2:**
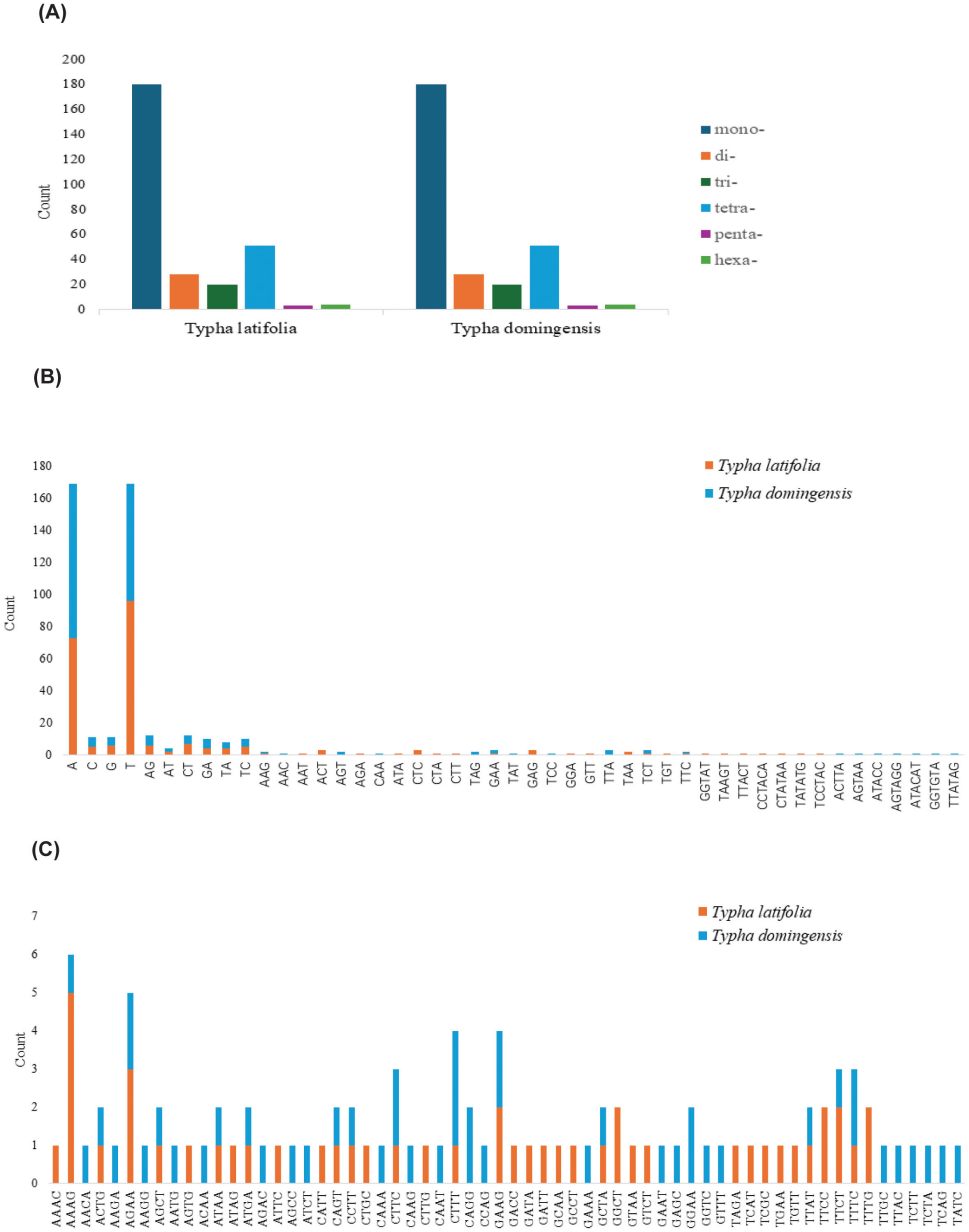
SSRs in two *Typha* species mitogenomes. **(A)** Count of six unit-sized SSRs. **(B)** Count of different unit components from mono- to hexa- except for tetra-nucleotide SSRs. **(C)** Count of different unit components of tetra-nucleotide SSRs.

**Figure 3:**
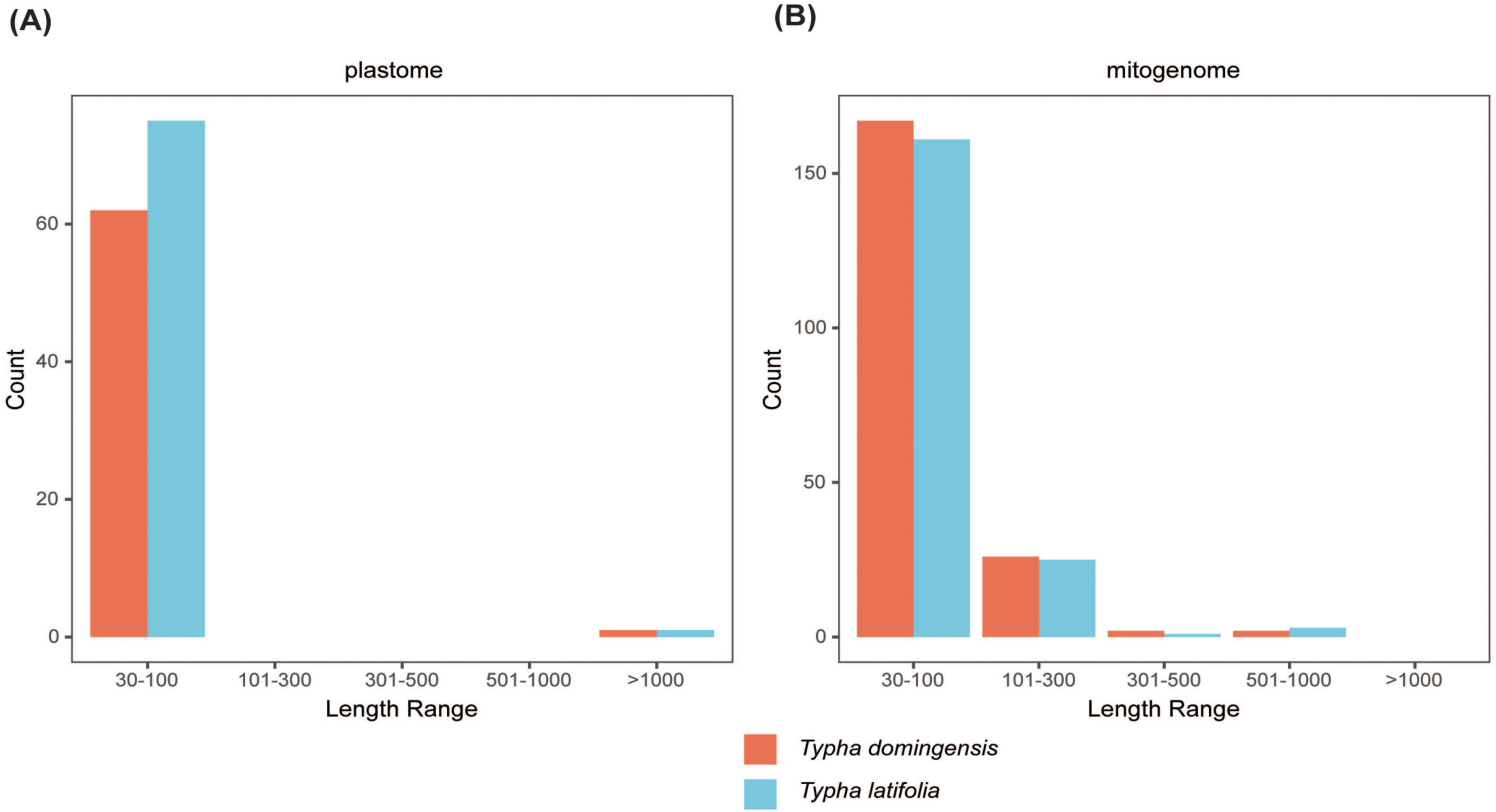
Length of dispersed repeats in two Typha species **(A)** plastomes and **(B)** mitochondrial genomes.

To clarify repeat sequence diversity, different types of repetitive sequences in two *Typha* species were investigated. Within the plastomes, the predominant SSRs feature a single-nucleotide repeat unit, with A/T repeats being most abundant. The proportion of A/T repeat units comprises 68.68% in *T. latifolia* and 44.8% in *T. domingensis* of all identified SSR repeats. However, the distribution of SSRs of different types in the mitogenomes is uniform. In both species, there are a total of 180 mononucleotide SSRs, 28 dinucleotide SSRs, 20 trinucleotide SSRs, 51 tetranucleotide SSRs, 3 pentanucleotide SSRs, and 4 hexanucleotide SSRs. Most of SSRs in the mitogenomes feature a tetranucleotide repeat unit, accounting for 55.0% and 59.0% of all repeat numbers. Additionally, homopolymer SSR A/T were also identified as prevalent SSR types (**Figure 2**). A total of 58 tandem repeats were identified in *T. latifolia* and 48 in *T. domingensis* within the plastomes (**Supplementary Tables S3**, **S5**) while 7 tandem repeats were found in *T. latifolia* and 6 in *T. domingensis* within the mitogenomes (**Supplementary Tables S4**, **S6**). Dispersed repeats across the plastomes and mitogenomes were identified as two types, forward and palindromic matches. While forward and palindromic repeats were predominant, one reverse repeat was also identified (**Supplementary Table S9**). In the plastomes, the most prevalent and longest repeats are palindromic, with the longest fragment measuring 26,930 bp in *T. latifolia* and 26,882 bp in *T. domingensis*. Additionally, 75 and 63 dispersed repeats were identified in the plastomes of *T. latifolia* and *T. domingensis*, respectively (**Supplementary Tables S8**, **S10**).The most abundant and longest repeats in the mitogenomes are forward repeats, with the longest fragment measuring 686 bp in both genomes. These forward repeats constitute 57.9% and 56.7% of the total repeats in the mitogenome. *T. latifolia* and *T. domingensis* mitogenomes possessed 190 and 198 dispersed repeats respectively (**Supplementary Tables S7**, **S9**). While repeats exceeding 1000 bp were observed in the plastomes, no such fragments were found in the mitogenomes (**Figure 3**). Additionally, a total of 32 shared codons encoding 18 amino acids (one was a stop codon, UGA) showed that RSCU exceeded 1 across all two *Typha* spp. (**Table 2**).

**Table 2:** RSCU of two *Typha* mitogenomes.

Amino acid	Codon	RSCU		Amino acid	Codon	RSCU		Amino acid	Codon	RSCU	
		*T. latifolia*	*T. domingensis*			*T. latifolia*	*T. domingensis*			*T. latifolia*	*T. domingensis*
Stop codon	UAA	0.87241	0.848	Ile	AUA	0.89953	0.886	Arg	**CGG**	1.06345	1.060
	UAG	0.90228	0.918		**AUC**	1.09670	1.092		CGU	0.83233	0.822
	**UGA**	1.22532	1.234	Lys	AAA	0.99819	0.976	Ser	**AGC**	1.09794	1.106
Ala	**GCU**	1.27494	1.292		**AAG**	1.00181	1.024		AGU	1.09794	0.894
	GCG	0.68727	0.704	Leu	CUA	0.77194	0.778		**UCA**	1.03773	1.041
	GCC	1.01538	0.999		**CUC**	1.01405	1.023		**UCC**	1.01934	1.031
	**GCA**	1.02241	1.005		CUG	0.78800	0.780		UCG	0.76555	0.721
Cys	UGU	0.94081	0.919		**CUU**	1.42601	1.420		**UCU**	1.17738	1.208
	**UGC**	1.05919	1.081		UUA	0.83122	0.826	Thr	**ACC**	1.05234	1.001
Asp	**GAU**	1.13582	1.166		**UUG**	1.16878	1.174		ACA	0.98173	1.061
	GAC	0.86418	0.834	Met	AUG	1.00000	1.000		ACG	0.74636	0.723
Glu	GAG	0.87607	0.860	Asn	AAC	0.88172	0.870		**ACU**	1.21957	1.214
	**GAA**	1.12393	1.140		**AAU**	1.11828	1.130	Val	**ACC**	1.05234	1.001
His	**UUU**	1.03912	1.017	Pro	**CCA**	1.07278	1.081		ACA	0.98173	1.061
	UUC	0.96088	0.983	Arg	**AGA**	1.13568	1.104		ACG	0.74636	0.723
Ile	**AUU**	1.00377	1.022		AGG	0.86432	0.896		**ACU**	1.21957	1.214
	AUA	0.89953	0.886		**CGA**	1.23431	1.257	Trp	UGG	1.00000	1.000
	**AUC**	1.09670	1.092		CGC	0.86990	0.861	Tyr	UAC	0.85324	0.828

Codons with RSCU > 1 are bold and underlined.

### Synteny and homologous sequences between mitogenome and plastome

**Figure 4:**
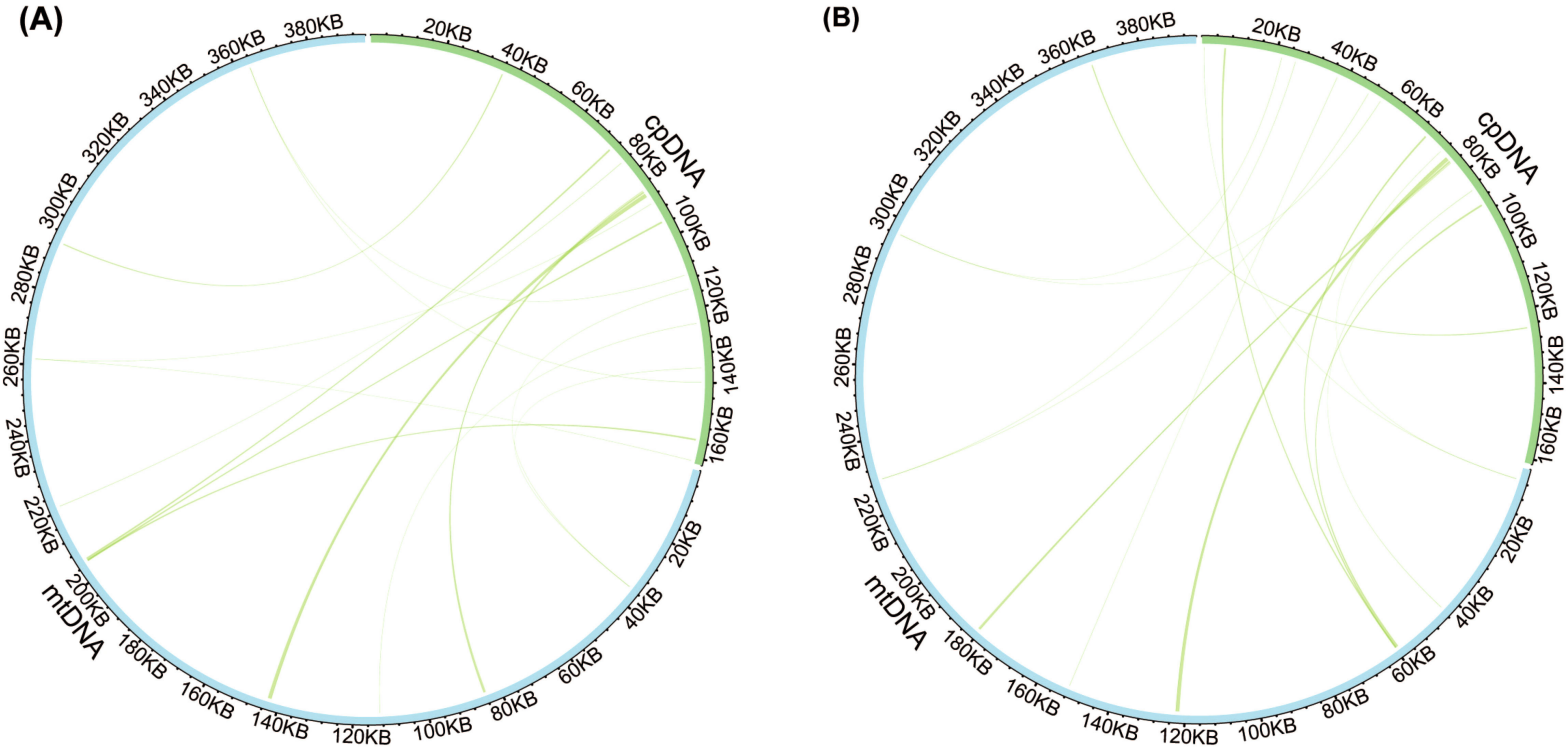
Comparison of the plastome and mitogenome of two *Typha* species. **(A)** Comparison of the plastome and mitogenome of *T. latifolia*. **(B)** Comparison of the plastome and mitogenome of *T. domingensis*. The blue and green outer arcs represent the mitogenome (mtDNA) and plastome (cpDNA), respectively, and the inner arcs show the homologous DNA fragments. The scale is shown on the outer arcs, with intervals of 20 kb.

**Figure 5:**
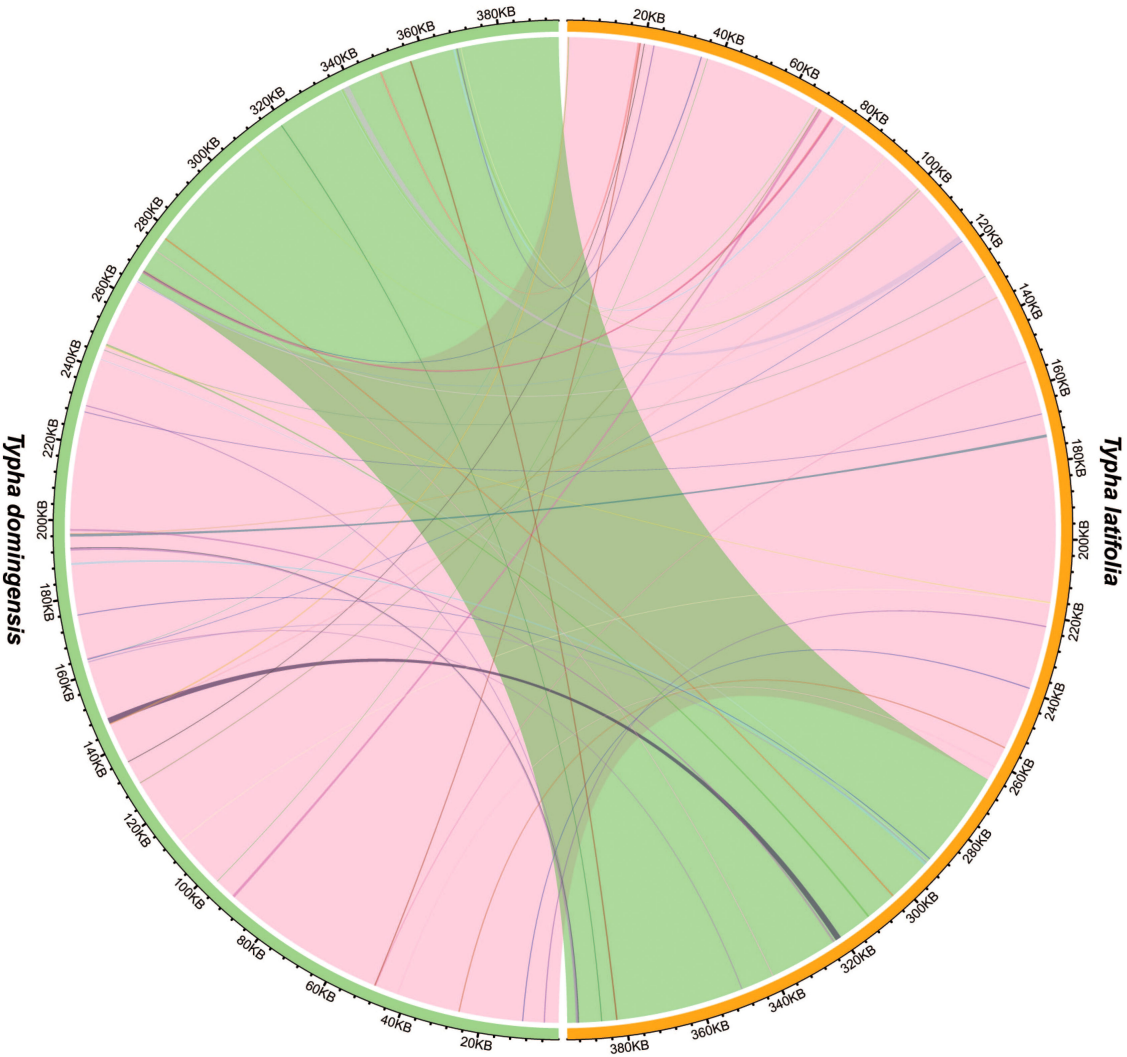
Interspecific mitogenomic synteny indicated by homologous regions. The orange and green outer arcs represent *T. latifolia* and *T. domingensis*, respectively, and the inner arcs show the homologous DNA fragments. The scale is shown on the outer arcs, with intervals of 20 kb.

Analysis of sequence similarity between the mitogenomes and plastomes of *T. latifolia* and *T. domingensis* revealed that 86,496 bp and 73,949 bp in the respective mitogenomes likely originated from past transfer from the plastomes, accounting for 21.9% and 18.7% of the respective mitogenome length (**Figure 4**). 16 homologous fragments between mitogenome and plastome were detected in both *Typha* species, with the longest spanning 959 bp in *T. latifolia* and 976 bp in *T. domingensis*. Eight transferred DNA fragments into mitogenome were identified in *T. latifolia* and *T. domingensis* that were located in plastome IR region half the total transfer fragments. In *T. latifolia* the transferred fragments encompass thirteen plastome genes (*rpl16, ycf2, rpl14, trnW-CCA, trnP-UGG, psbD, rps8, clpP1, trnN-GUU, rrn23, rpl16, ndhE*, and *trnH-GUG*) and six mitochondrial genes (*trnW-CCA, trnP-UGG, trnN-GUU, trnH-GUG, rrn26*, and *trnH-GUG*). In *T. domingensis*, the homologous fragments include ten plastome genes (*rps12, cemA, clpP1, rpl2, ndhI, ycfI, trnL-UAA, rpl20, psbC, psbA*) and four mitochondrial genes (*trnW-CCA, trnP-UGG, trnN-GUU*, and *trnH-GUG*). The synteny analysis of the entire mitogenome in two *Typha* species revealed extensive interspecific large homologous regions, with two crosses observed. In addition, small homologous regions could be found across the entire mitogenome, which revealed high compositional and low structural conservation (**Figure 5**).

### Phylogenetic analysis

**Figure 6:**
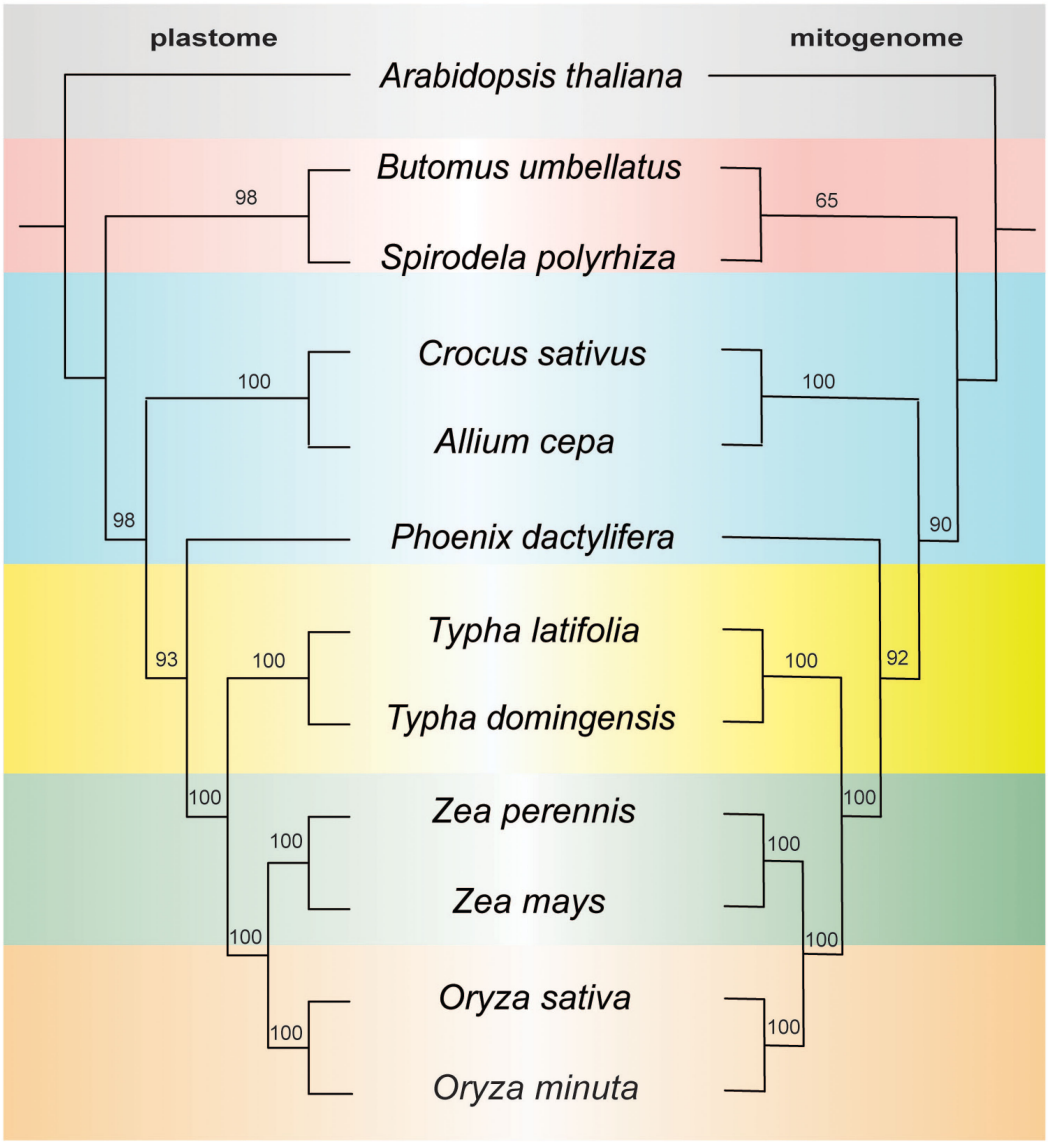
ML tree based on 77 shared plastid genes (left) and 24 shared mitochondrial genes (right). The numbers near nodes indicated the bootstrap value.

To investigate the evolutionary patterns of organellar genomes in two *Typha* species, we performed a phylogenetic analysis using the organelle genomes of these species along with 10 related species (*Arabidopsis thaliana* was chosen as outgroup for comparison). Our analysis involved the nucleotide sequences of 77 common genes (*accD, atpA, atpB, atpE, atpF, atpH, atpI, ccsA, cemA, clpP1, infA, matK, ndhA, ndhB, ndhC, ndhD, ndhE, ndhF, ndhG, ndhH, ndhI, ndhK, pafI, petA, petB, petD, petG, petL, petN, psaA, psaB, psaC, psaI, psaJ, psbA, psbB, psbC, psbD, psbE, psbF, psbH, psbI, psbJ, psbK, psbL, psbM, psbT, psbZ, rbcl, rpl2, rpl14, rpl16, rpl20, rpl22, rpl23, rpl32, rpl33, rpl36, rpoA, rpoB, rpoC1, rpoC2, rps2, rp23, rps4, rps7, rps8, rps11, rps12, rps14, rps15, rps16, rps18, rps19, ycfI, and ycfII*) for the plastome-based phylogenetic analysis and 24 common genes (*atp1, atp4, atp6, atp8, atp9, ccmB, ccmC, cob, cox1, cox2, cox3, nad1, nad2, nad4, nad4L, nad5, nad6, nad7, nad9, rpl6, rps1, rps3, rps4, rps12*) for the mitogenome-based analysis. Notably, the plastome tree exhibited full support for all nodes, except for three nodes with a bootstrap value of 93, 98, and 98. The mitogenome tree showed one node with weak support with a bootstrap value of 65, all others had strong bootstrap support. Despite differences in clade support the topology was identical between the datasets and the two *Typha* species formed a monophyletic clade (**Figure 6**). Based on the data of mitogenome and plastome, the genus *Typha* was closer to the Poaceae clade, which includes maize and rice, consistent with previously published reports (Guisinger et al., 2010).

## Discussion

*Typha* is an ecologically crucial lineage of plants in marshes and wetlands globally, contributing significantly to bioremediation, ecological functioning, and serving as a source of biofuel materials (Bonanno and Cirelli, 2017). The assembly and annotation of the *Typha* organellar genomes, is expected to serve as a significant tool in wetland management (Bansal et al., 2019). Here, we present the first published organellar genome assemblies from the Typhaceae family. In the present study, the mitogenomes of *T. latifolia* (395,136bp) and *T. domingensis* (395,140bp) are more than twice the size of the respective plastomes (161,545bp and 161,230bp). In plants, the mitogenome is nearly always considerably larger than the plastome (Ye Ning et al., 2017; Cheng et al., 2021). It is also common for some part of the mitogenome to be homologous to the plastome (Hazkani-Covo et al., 2010; Smith, 2011), which are known as so-called intracellular gene transfer (IGT). Persistent IGTs continuously reshape and increase the complexity of plant mitogenomes. For example, these homologous regions within mitogenomes can be hotspots for recombination, possibly generating chimeric open reading frame (ROFs) with new functions (Wang et al., 2024b). Mitochondrial plastid DNAs (MTPTs) occurred at least 300 MYA and been described for numerous species. However, the model of MTPTs was not clear. Early previously reported that cpDNA transfers were randomly and likely occurred at any positions of the cpDNAs (Wang et al., 2007), while transfer hot spots and cold spots chloroplast genome were exist based on the frequency of genes (Wang et al., 2018). We found 16 fragments in the mitogenome of two *Typha* species, representing approximately 6.6% of the mitogenome, and there are eight transferred DNA fragments were identified that were located in *T. latifolia* and *T. domingensis* plastome IR region half the total transfer fragments. This finding was consistent with previously reported that IR region was highest coverage of MTPTs (Nhat Nam et al., 2024). In comparing the genome size between organelles, the difference between plastomes was greater than between mitogenomes. Specifically, the *T. latifolia* plastome was 315 bp larger than that of *T. domingensis* primarily due to the expansion and contraction of the single-copy (SC) and inverted repeat (IR) boundaries. In contrast, the mitogenome of *T. latifolia* was 4 bp smaller than that of *T. domingensis*. Similar variations in plastome lengths due to the expansion and contraction of SC/IR boundaries have been observed in other plant species, as reported by (Jansen and Ruhlman, 2012; Ruhlman and Jansen, 2014; Mower and Vickrey, 2018).

Plant mitogenomes are commonly described as circular structures, similar to the circular chromosomes present in bacteria and animal mitochondria. However, numerous studies have demonstrated that the basic circular model of genome structure, which is applicable to the majority of animal species, is inadequate for comprehending plant mitochondria, which can possess multiple circular replicons, branched, linear, or mixed forms of genomic structure. For example, the *Silene noctiflora* mitogenome is arranged into multiple circular chromosomes; thus, one ring cannot rule them all based on mapping to a circular reference (Sloan, 2013; Wu et al., 2015). Both *T. latifolia* and *T. domingensis* mitogenomes were assembled into a single circular structure which may explain the similarity in size between the two species examined here. However, future studies should examine more species and investigate the possibility of tissue-specific mitogenome variation, as has been observed in some plants. Here, *T. domingensis* demonstrates higher copy numbers for *trnI-GAU* and *rps12* in comparison to *T. latifolia*. The two *Typha* mitogenomes shared 59 unique genes, including 39 PCGs, three rRNA genes, and 17 tRNA genes, which are highly similar to those of other species such as *Oryza sativa* (Notsu et al., 2002) and *Arabidops*is *thaliana* (Unseld et al., 1997). Since the mitogenome is in control of oxidative phosphorylation, the fundamental energy production required for living activities, the conservation of gene content throughout several plant lineages suggests that the mitogenome has a stable function (Mackenzie and McIntosh, 1999). A closer relationship between *T. latifolia* and *T. domingensis* within *Typha* was supported by the phylogenetic analysis that followed, which was based on organellar genes. This was consistent with the synteny pattern, which showed that there were more bulks of synteny between these two species, indicating a more similar sequence component and arrangement.

Phylogenomic analysis using our assembled mitogenomes and plastomes showed congruent results. This congruence between plastome and mitogenome topologies has also been found in other plant taxa, such as *Saposhnikovia* and *Moraceae*, where both organellar genomes exhibit similar evolutionary histories (Ni et al., 2022) (Lai et al., 2022). However, it is noteworthy that the plastome tree showed higher support for certain clades compared to the mitogenome tree, suggesting that while the overall topologies are congruent, there may be differences in the rates of evolution between organelles. Plants mitogenomes exhibit extensive variation in size and structure, ranging from approximately 66 kb in *Viscum scurruloideum* to about 12 Mb in *Larix sibirica* (Wang et al., 2024b). Complex mitogenomes contain a large number of repetitive sequences, as seen in the mitochondrial genome of *Panax notoginseng*, where 64 pairs of recombination-mediated repeats form a multi-molecular structure comprising a “master circle” that interconverts with numerous “subgenomic circles” (Yang et al., 2023). The types of repetitive sequences found in plant mitochondrial genomes include inverted repeats, tandem repeats, non-tandem repeats, and large repeats (Wynn and Christensen, 2019). These repetitive sequences are involved in genome replication, recombination, insertion, and deletion, playing a crucial role in the evolution of mitochondrial genome size and structure in plants (Wang et al., 2024a) Here, *T. latifolia* and *T. domingensis* mitogenomes were assembled into circular structure, with size of 395,136 bp and 395,140 bp. No long repetitive sequences greater than 1,000 bp were detected in the two mitogenomes, which may explain why these genomes were assembled into simple circular structures with a small size.

## Conclusions

The mitogenomes of two *Typha* species were described for the first time in this work, and their plastomes were constructed using the same sequencing data set. Here, *T. latifolia* and *T. domingensis* mitogenomes were assembled into circular structure, with size of 395,136 bp and 395,140 bp. 39 protein-coding genes (PCGs) were annotated in the each *Typha* species mitogenome. Congruent trees with 10 related species were displayed by phylogenomic analysis using the assembled plastome and mitogenome. Based on the data of mitogenome and plastome, the genus *Typha* was closer to the Poaceae clade, which includes maize and rice. Through sequence comparisons between the mitogenome and plastome, we were able to identify twelve mitochondrial DNA fragments that were similar to those found in the plastome. This study provides valuable information to understand the coordinated evolution of the plastome and the mitogenomes of plant species belonging to the family Typhaceae and monocots in general.

## Data Availability

The original contributions presented in the study are included in the article/**Supplementary Material**. Further inquiries can be directed to the corresponding authors.
