# Supplementary material for: Organelle genome assembly, annotation, and comparative analyses of Typha latifolia and T. domingensis: two keystone species for wetlands worldwide
Source: Front Plant Sci. 2024 Dec 5;15:1484531. doi: 10.3389/fpls.2024.1484531 (PMC11655213; doi:10.3389/fpls.2024.1484531)
Supplement: Supplementary file 1 [file DataSheet1.pdf]

Table and figure legend:

Table S1 Gene compositions of *Typha latifolia* plastome.

Category of genes	Group of genes	Name of genes
	rRNA	*rrn*4.5S (×2), *rrn*5S (×2), *rrn*16S (×2), *rrn*23S* (×4)
	tRNA	*trnH-GUG (×2), trnI-CAU (×2), trnL-UAG, trnL-UAA*, trnI-GAU*(×4), trnL-CAA (×2), trnV-GAC (×2), trnV-UAC* (×2), trnA-UGC* (×2), trnR-ACG (×2), trnR-UCU, trnN-GUU (×2), trnP-UGG, trnW-CCA, trnM-CAU (×2), trnF-GAA, trnT-UGU, trnT-GGU, trnS-GGA, trnS-UGA, trnS-GCU, trnfM-CAU, trnG-GCC* (×2), trnG-UCC*(×2), trnE-UUC, trnY-GUA, trnD-GUC, trnC-GCA, trnQ-UUG, trnK-UUU**
photosynthesis	Subunits of ATP synthase	*atp*A, *atp*B, *atp*E, *atp*F* (×2), *atp*H, *atp*I
	Subunits of photosystem II	*psb*A, *psb*B, *psb*C (×2), *psb*D, *psb*E, *psb*F, *psb*H, *psb*I, *psb*J, *psb*K, psbN, *psb*L, *psb*M, *psb*T, *psb*Z
	Subunits of cytochrome b/f complex	*pet*A, *pet*G, *pet*L, *pet*N, petB*, petD*
	Subunits of photosystem I	*psa*A, *psaB*, *psa*C, *psa*I, *psa*J
	Subunit of rubisco	*rbc*L
	Subunits of NADH-dehydrogenase	*ndh*A* (×2), *ndh*B* (×2), *ndh*C, *ndh*D, *ndh*E, *ndh*F, *ndh*G, *ndh*H, *ndh*I, *ndh*J, *ndh*K (×2)
Self-replication	Large subunit of ribosome	*rpl*14, *rpl16**, *rpl*2* (×4), *rpl*20, *rpl*22, *rpl*23 (×2), *rpl*32, *rpl*33, *rpl*36

	DNA dependent RNA polymerase	*rpo*A, *rpo*B, *rpo*C1* (×2), *rpo*C2
	Small subunit of ribosome	*rps*11, *rps*12*, *rps*14, *rps*15, *rps*16* (×2), *rps*18, *rps*19 (×2), *rps*2, *rps*3, *rps*4, *rps*7 (×2), *rps*8
	Subunit of Acetyl-CoA-carboxylase	*acc*D
Other genes	c-type cytochrome synthesis gene	*ccs*A
	Envelop membrane protein	*cem*A
	Protease	*clp*P ** (×2)
	Translational initiation	*inf*A
	Maturase	*matK*
Unknown	Conserves open reading frames	*ycf*1 (×2), *ycf*2 (×2), *ycf3*** (×2), *ycf4*

Notes: * one intron, ** two intron; Gene (2): Number of copies of multi-copy genes.

Table S2 Gene compositions of the *Typha* domingensis plastome.

Category of genes	Group of genes	Name of genes
	rRNA	*rrn*4.5S (×2), *rrn*5S (×2), *rrn*16S (×2), *rrn*23S* (×2)
	tRNA	trnH-GUG (×2), trnI-CAU (×2), trnI-GAU* (×4), trnL-CAA (×2), trnL-UAG, trnL-UAA*, trnV-GAC (×2), trnV-UAC* (×2), trnA-UGC* (×2), trnR-ACG (×2), trnN-GUU (×2), trnP-UGG, trnW-CCA, trnM-CAU (×2), trnF-GAA, trnT-UGU, trnT-GGU, trnS-GGA, trnS-UGA, trnS-GCU, trnfM-CAU, trnG-GCC* (×2), trnG-UCC* (×2), trnE-UUC, trnY-GUA, trnD-GUC, trnC-GCA, trnR-UCU, trnQ-UUG, trnK-UUU*
photosynthesis	Subunits of ATP synthase	*atp*A, *atp*B, *atp*E, *atp*F *, *atp*H, *atp*I

	Subunits of photosystem II	*psb*A, *psb*B, *psb*C, *psb*D, *psb*E, *psb*F, *psb*H, *psb*I, *psb*J, *psb*K, psbN, *psb*L, *psb*M, *psb*T, *psbZ*
	Subunits of cytochrome b/f complex	*pet*A, *pet*G, *pet*L, petD*, petB*, *pet*N
	Subunits of photosystem I	*psa*A, *psaB*, *psa*C, *psa*I, *psa*J
	Subunit of rubisco	*rbc*L
	Subunits of NADH-dehydrogenase	*ndh*A*, *ndh*B* (×2), *ndh*C, *ndh*D, *ndh*E, *ndh*F, *ndh*G, *ndh*H, *ndh*I, *ndh*J, *ndh*K (×2)
Self-replication	Large subunit of ribosome	*rpl*14, rpl16*, *rpl2* * (×2), *rpl*20, *rpl*22, *rpl*23* (×2), *rpl*32, *rpl*33, *rpl*36,
	DNA dependent RNA polymerase	*rpo*A, *rpo*B, *rpo*C1*, *rpo*C2
	Small subunit of ribosome	*rps*11, *rps*12* (×2), *rps*14, *rps*15, *rps*16*, *rps*18, *rps*19 (×2), *rps*2, *rps*3, *rps*4, *rps*7 (×2), *rps*8
	Subunit of Acetyl-CoA-carboxylase	*acc*D
Other genes	c-type cytochrome synthesis gene	*ccs*A
	Envelop membrane protein	*cem*A
	Protease	*clp*P **
	Translational initiation	*inf*A
	Maturase	*matK*
Unknown	Conserves open reading frames	*ycf*1 (×2), *ycf*2 (×2), *ycf3*** (×2), *ycf4*

Notes: * one intron, ** two intron; Gene (2): Number of copies of multi-copy genes.

Table S3 Tandem repeats in the *T. latifolia* plastome.

Indices	Period Size	Copy Number	Consensus Size	Percent Matches	Percent Indels	Score	A	C	G	T	Entropy (0-2)
5445--5469	13	1.9	13	100	0	50	8	16	4	72	1.24
6496--6524	14	2.1	14	100	0	58	37	6	20	34	1.80
8728--8758	15	2.1	15	100	0	62	45	6	0	48	1.28

8799--8840	20	2.1	20	100	0	84	30	14	4	50	1.63
8840--8866	13	2.1	13	100	0	54	29	14	0	55	1.40
8953--9020	30	2.4	27	90	9	100	63	4	0	32	1.14
15397--15426	13	2.3	13	100	0	60	53	6	6	33	1.53
15447--15486	19	2.0	20	90	4	64	57	0	7	35	1.27
15458--15527	36	2.0	35	91	2	113	51	0	12	35	1.40
15595--15629	17	2.1	17	94	0	61	31	14	5	48	1.67
17908--17937	15	2.1	15	93	6	53	26	6	20	46	1.75
22195--22224	14	2.1	14	93	0	51	23	6	16	53	1.66
32134--32164	15	2.1	15	100	0	62	48	12	6	32	1.67
33197--33237	18	2.3	18	95	4	75	21	0	9	68	1.18
34298--34335	8	4.6	8	90	6	58	44	10	5	39	1.61
34298--34335	17	2.3	16	90	4	58	44	10	5	39	1.61
38910--38948	19	2.1	19	90	0	60	38	12	7	41	1.72
48855--48891	14	2.6	14	95	0	65	48	0	5	45	1.25
49428--49529	37	2.8	37	98	0	195	50	0	9	40	1.36
51617--51645	11	2.6	11	100	0	58	0	20	0	79	0.74
51697--51747	25	2.0	25	100	0	102	49	3	19	27	1.66
54377--54405	14	2.1	14	93	6	51	20	6	10	62	1.50
54441--54475	17	2.1	17	100	0	70	25	5	11	57	1.56
55961--55992	15	2.1	15	94	0	55	65	9	6	18	1.42
56008--56036	12	2.3	13	94	5	51	24	3	24	48	1.66
60394--60423	9	3.2	9	90	9	51	53	10	10	26	1.66
61611--61643	15	2.2	15	100	0	66	42	6	21	30	1.77
63033--63073	16	2.6	16	96	3	75	43	0	0	56	0.99
63070--63129	27	2.2	28	90	3	95	38	15	3	43	1.63

66383--66409	14	1.9	14	100	0	54	37	25	7	29	1.83
67525--67559	18	1.9	18	100	0	70	40	20	5	34	1.76
68178--68217	20	2.0	20	100	0	80	55	5	0	40	1.22
69955--69987	16	2.1	16	100	0	66	54	0	6	39	1.25
72075--72116	21	2.0	21	100	0	84	33	0	28	38	1.58
72418--72457	16	2.5	16	95	0	71	52	7	0	40	1.30
85760--85804	15	3.0	15	100	0	90	20	0	6	73	1.05
87482--87511	15	2.0	15	100	0	60	40	6	0	53	1.27
94449--94531	21	3.8	21	91	4	121	14	27	10	46	1.78
94470--94538	21	3.3	21	95	0	120	13	31	8	46	1.73
96895--96971	24	3.2	24	96	0	145	32	9	24	33	1.87
99109--99154	22	2.1	22	95	0	83	63	2	17	17	1.42
99943--99993	25	2.0	25	100	0	102	39	5	19	35	1.76
105174--105198	12	2.1	12	100	0	50	16	24	28	32	1.96
118595--118621	13	2.1	13	100	0	54	29	7	14	48	1.71
119559--119586	14	2.0	14	100	0	56	21	21	0	57	1.41
123315--123352	18	2.1	18	90	0	58	44	7	13	34	1.72
123625--123662	19	2.0	19	100	0	76	31	0	10	57	1.32
123825--123892	28	2.5	28	90	2	102	39	7	1	51	1.39
130030--130069	20	2.0	20	100	0	80	25	10	15	50	1.74
130139--130179	15	2.9	15	92	7	68	46	0	0	53	1.00
130143--130187	23	1.9	23	90	4	72	46	0	2	51	1.13
145479--145503	12	2.1	12	100	0	50	32	28	24	16	1.96
150684--150734	25	2.0	25	100	0	102	35	19	5	39	1.76
150684--150742	25	2.4	25	91	5	84	37	16	6	38	1.76
151523--151568	22	2.1	22	95	0	83	17	17	2	63	1.42

153706--153782	24	3.2	24	96	0	145	33	24	9	32	1.87
156139--156207	21	3.3	21	95	0	120	46	8	31	13	1.73
156146--156228	21	3.8	21	91	4	121	46	10	27	14	1.78

Parameters: Total length (Period size * Copy number) >= 30 and Percent Matches >= 90%

Table S4 Tandem repeats in the *T. latifolia* mitogenome.

Indices	Period Size	Copy Number	Consensus Size	Percent Matches	Percent Indels	Score	A	C	G	T	Entropy (0-2)
80588--80629	21	2.0	21	85	0	57	28	23	23	23	2.00
129241--129270	12	2.5	12	94	0	51	0	26	10	63	1.26
134777--134825	18	2.7	18	81	9	55	24	22	32	20	1.98
153016--153058	20	2.1	20	82	4	50	25	16	9	48	1.75
277062--277107	23	2.0	23	95	0	83	23	13	28	34	1.92
322004--322120	42	2.8	42	98	0	225	35	25	13	25	1.93
371920--371944	12	2.1	12	100	0	50	28	32	16	24	1.96

Table S5 Tandem repeats in the *T. domingensis* plastome.

Indices	Period Size	Copy Number	Consensus Size	Percent Matches	Percent Indels	Score	A	C	G	T	Entropy (0-2)
5320--5387	21	3.3	21	91	4	111	13	32	8	45	1.74
7741--7817	24	3.2	24	96	0	145	32	9	24	33	1.87
9953--9998	22	2.1	22	95	0	83	63	2	17	17	1.42
10786--10836	25	2.0	25	100	0	102	39	5	19	35	1.76
16012--16036	12	2.1	12	100	0	50	16	24	28	32	1.96
31399--31438	20	2.0	20	100	0	80	50	15	10	25	1.74
37803--37840	19	2.0	19	100	0	76	57	10	0	31	1.32
41871--41898	14	2.0	14	100	0	56	57	0	21	21	1.41

42832--42858	13	2.1	13	100	0	54	48	14	7	29	1.71
56231--56255	12	2.1	12	100	0	50	32	28	24	16	1.96
61431--61481	25	2.0	25	100	0	102	35	19	5	39	1.76
61431--61489	25	2.4	25	91	5	84	37	16	6	38	1.76
62269--62314	22	2.1	22	95	0	83	17	17	2	63	1.42
64450--64526	24	3.2	24	96	0	145	33	24	9	32	1.87
66880--66947	21	3.3	21	91	4	111	45	8	32	13	1.74
73887--73916	15	2.0	15	100	0	60	53	0	6	40	1.27
75588--75629	14	3.0	14	100	0	84	71	7	0	21	1.09
88903--88942	16	2.5	16	95	0	71	40	0	7	52	1.30
89243--89284	21	2.0	21	100	0	84	38	28	0	33	1.58
91362--91394	16	2.1	16	100	0	66	39	6	0	54	1.25
93131--93170	20	2.0	20	100	0	80	40	0	5	55	1.22
93786--93820	18	1.9	18	100	0	70	34	5	20	40	1.76
94932--94958	14	1.9	14	100	0	54	29	7	25	37	1.83
98256--98296	16	2.6	16	96	3	75	56	0	0	43	0.99
99685--99717	15	2.2	15	100	0	66	30	21	6	42	1.77
100902--100930	9	3.0	10	90	10	51	27	10	10	51	1.68
105276--105304	12	2.3	13	94	5	51	48	24	3	24	1.66
106835--106869	17	2.1	17	100	0	70	57	11	5	25	1.56
106905--106933	14	2.1	14	93	6	51	62	10	6	20	1.50
109562--109612	25	2.0	25	100	0	102	27	19	3	49	1.66
111789--111897	41	2.7	41	92	2	175	41	8	0	50	1.32
112417--112453	14	2.6	14	95	0	65	45	5	0	48	1.25
126943--126980	17	2.3	16	90	4	58	39	5	10	44	1.61
128041--128081	18	2.3	18	95	4	75	68	9	0	21	1.18

129102--129132	15	2.1	15	100	0	62	32	6	12	48	1.67
139025--139054	14	2.1	14	93	0	51	53	16	6	23	1.66
143305--143334	15	2.1	15	93	6	53	46	20	6	26	1.75
145607--145641	17	2.1	17	94	0	61	48	5	14	31	1.67
145709--145778	36	2.0	35	91	2	113	35	12	0	51	1.40
145760--145812	19	2.8	19	94	5	90	37	9	0	52	1.34
145829--145858	13	2.3	13	100	0	60	33	6	6	53	1.53
151505--151535	15	2.1	15	93	0	53	51	0	0	48	1.00
152225--152292	30	2.4	27	90	9	100	32	0	4	63	1.14
152379--152405	13	2.1	13	100	0	54	55	0	14	29	1.40
152405--152446	20	2.1	20	100	0	84	50	4	14	30	1.63
152487--152517	15	2.1	15	100	0	62	48	0	6	45	1.28
154716--154744	14	2.1	14	100	0	58	34	20	6	37	1.80
155770--155794	13	1.9	13	100	0	50	72	4	16	8	1.24

Parameters: Total length (Period size * Copy number) >= 30 and Percent Matches >= 90%

Table S6 Tandem repeats in the *T. domingensis* mitogenome.

Indices	Period Size	Copy Number	Consensus Size	Percent Matches	Percent Indels	Score	A	C	G	T	Entropy (0-2)
129919--129967	18	2.7	17	81	9	53	20	32	22	24	1.98
135474--135503	12	2.5	12	94	0	51	63	10	26	0	1.26
184115--184156	21	2.0	21	85	0	57	23	23	23	28	2.00
287939--287963	12	2.1	12	100	0	50	24	16	32	28	1.96
337764--337880	42	2.8	42	98	0	225	25	13	25	35	1.93

382777--382822	23	2.0	23	95	0	83	34	28	13	23	1.92

Table S7 Dispersed repeats in the *T. latifolia* mitogenome.

The repeat length of the first part	The starting site of the first part	Matching direction	The repeat length of the second part	The starting site of the second part	interval distance of repeats	E-value
686	116795	F	686	32045	0	0.00E+0
658	68209	P	658	17295	0	0.00E+0
524	75684	F	524	291535	0	0.00E+00
337	117588	F	337	321286	-3	9.56E-185
272	303255	F	272	382218	0	7.63E-154
240	117733	F	240	321431	-3	8.64E-127
220	153389	P	220	216382	-1	1.02E-119
199	222463	F	199	260963	0	6.80E-110
202	340441	P	202	386175	-1	6.44E-109
195	101399	P	195	291132	0	1.74E-107
199	72250	F	199	392702	-1	4.06E-107
203	238862	F	203	255385	-2	4.90E-107
191	224	F	191	118254	0	4.46E-105
191	238874	F	191	255397	0	4.46E-105
191	340452	P	191	386175	0	4.46E-105
199	71968	F	199	392432	-3	2.38E-102
182	71985	F	182	392449	0	1.17E-99
184	117891	F	184	321589	-3	2.01E-93
179	117932	F	179	321630	-3	1.90E-90
161	34832	F	161	392281	0	5.14E-87

153	71766	F	153	392138	0	3.37E-82
155	117974	F	155	321672	-3	3.46E-76
141	100569	F	141	349129	-1	2.39E-72
133	22355	P	133	290970	0	3.70E-70
127	68082	F	127	135307	0	1.52E-66
130	66969	F	130	322734	-2	1.79E-63
124	19841	P	124	129070	-1	3.61E-62
116	18816	F	116	216266	-2	3.82E-55
116	36511	F	116	167449	-2	3.82E-55
95	199884	F	95	246801	0	2.80E-47
95	263011	P	95	323919	0	2.80E-47
93	19872	P	93	129070	0	4.48E-46
90	71612	P	90	183130	0	2.87E-44
89	315422	F	89	349259	0	1.15E-43
90	18842	F	90	216292	-1	7.74E-42
84	1644	F	84	75669	0	1.17E-40
94	312	P	94	100807	-3	4.05E-40
94	100807	P	94	118342	-3	4.05E-40
83	25739	F	83	344776	0	4.69E-40
93	89127	P	93	289923	-3	1.57E-39
82	18850	F	82	216300	0	1.88E-39
90	100815	P	90	118338	-3	9.09E-38
82	18856	P	82	394871	-1	4.62E-37
81	235716	P	81	325920	-1	1.83E-36
81	289113	P	81	342314	-1	1.83E-36
76	100634	F	76	349194	0	7.69E-36
74	60619	F	74	137549	0	1.23E-34
81	18479	F	81	311680	-2	2.19E-34
73	72593	F	73	73035	0	4.92E-34

80	117472	F	80	321173	-2	8.55E-34
76	153538	P	76	216377	-1	1.75E-33
76	216306	P	76	394877	-1	1.75E-33
75	322003	F	75	322045	-1	6.92E-33
74	30770	F	74	232736	-1	2.73E-32
77	160391	F	77	321561	-2	5.06E-32
80	18208	F	80	311412	-3	6.67E-32
69	1659	F	69	291535	0	1.26E-31
78	18222	F	78	311426	-3	9.87E-31
71	24283	F	71	95594	-1	1.68E-30
77	117863	F	77	160391	-3	3.80E-30
66	72171	F	66	392623	0	8.07E-30
66	286368	F	66	300677	0	8.07E-30
68	18492	F	68	311693	-1	1.03E-28
67	18740	F	67	216190	-1	4.05E-28
62	301117	F	62	301698	0	2.06E-27
69	140549	P	69	239489	-2	2.66E-27
61	101	P	61	270064	0	8.26E-27
71	94966	P	71	227788	-3	1.22E-26
71	118087	F	71	321785	-3	1.22E-26
59	54749	P	59	191832	0	1.32E-25
62	145655	P	62	222161	-1	3.84E-25
58	75776	F	58	221538	0	5.29E-25
58	221538	F	58	291627	0	5.29E-25
57	18503	F	57	311704	0	2.11E-24
67	67284	F	67	322483	-3	2.61E-24
56	73777	P	56	255569	0	8.46E-24
65	101337	P	65	135489	-3	3.80E-23
64	118076	F	64	321774	-3	1.45E-22

53	21079	F	53	362298	0	5.41E-22
56	101346	P	56	135489	-1	1.42E-21
59	89065	P	59	290019	-2	2.03E-21
55	89165	P	55	289923	-1	5.58E-21
54	239081	P	54	360672	-1	2.19E-20
53	18159	P	53	129181	-1	8.61E-20
59	89053	P	59	290031	-3	1.16E-19
49	237852	P	49	309906	0	1.39E-19
46	202502	P	46	210287	0	8.87E-18
49	140569	P	49	239489	-1	2.04E-17
49	160419	F	49	321589	-1	2.04E-17
48	94989	P	48	227788	-1	7.98E-17
48	118152	F	48	321855	-1	7.98E-17
44	6642	F	44	117547	0	1.42E-16
43	145674	P	43	222161	0	5.68E-16
46	18254	F	46	311458	-1	1.22E-15
51	75497	P	51	379768	-3	4.87E-15
51	89113	P	51	289979	-3	4.87E-15
41	289153	P	41	342314	0	9.08E-15
47	75775	P	47	312401	-2	2.16E-14
47	291626	P	47	312401	-2	2.16E-14
40	140578	P	40	239489	0	3.63E-14
40	322658	F	40	351353	0	3.63E-14
43	118089	F	43	393424	-1	7.32E-14
46	118112	F	46	321810	-2	8.26E-14
46	221538	P	46	312401	-2	8.26E-14
39	52798	F	39	177786	0	1.45E-13
47	52729	F	47	326194	-3	9.71E-13
41	93201	P	41	215710	-1	1.12E-12

41	117511	F	41	321212	-1	1.12E-12
44	1565	P	44	218669	-2	1.21E-12
37	178	F	37	118201	0	2.32E-12
46	18411	F	46	311621	-3	3.63E-12
40	143683	P	40	346679	-1	4.36E-12
36	17343	P	36	342417	0	9.30E-12
36	66965	P	36	364273	0	9.30E-12
42	117898	F	42	160426	-2	1.76E-11
35	18903	P	35	394871	0	3.72E-11
35	177779	F	35	201010	0	3.72E-11
44	75513	P	44	379759	-3	5.07E-11
38	41213	F	38	326831	-1	6.63E-11
41	22065	P	41	159904	-2	6.70E-11
41	229605	F	41	276730	-2	6.70E-11
34	7527	P	34	156717	0	1.49E-10
34	68329	F	34	181975	0	1.49E-10
34	173409	P	34	181975	0	1.49E-10
34	222665	P	34	222665	0	1.49E-10
40	252668	P	40	252668	-2	2.55E-10
40	321787	F	40	393424	-2	2.55E-10
37	67234	F	37	322433	-1	2.58E-10
42	174	F	42	321899	-3	7.04E-10
42	81388	F	42	215748	-3	7.04E-10
42	89091	P	42	290010	-3	7.04E-10
39	128265	P	39	271937	-2	9.69E-10
32	101370	P	32	135489	0	2.38E-09
38	68364	P	38	340848	-2	3.68E-09
38	118200	F	38	321902	-2	3.68E-09
38	173370	F	38	340848	-2	3.68E-09

35	258680	F	35	325218	-1	3.91E-09
31	17213	P	31	34476	0	9.52E-09
31	78413	F	31	170316	0	9.52E-09
40	213150	P	40	253011	-3	9.69E-09
30	162740	F	30	342401	0	3.81E-08
30	222512	P	30	256367	0	3.81E-08
30	256367	P	30	261012	0	3.81E-08
30	262783	P	30	262783	0	3.81E-08
33	179846	F	33	372140	-1	5.89E-08
33	322003	F	33	322087	-1	5.89E-08
35	179422	F	35	355451	-2	1.99E-07
32	78087	F	32	169971	-1	2.29E-07
37	22351	F	37	96993	-3	4.88E-07
31	52745	F	31	326210	-1	8.86E-07
36	22275	P	36	362443	-3	1.79E-06
33	130772	F	33	222192	-2	2.83E-06
30	52798	F	30	201017	-1	3.43E-06
30	118208	F	30	321910	-1	3.43E-06
30	119647	F	30	120002	-1	3.43E-06
30	216353	P	30	394876	-1	3.43E-06
35	202093	F	35	386755	-3	6.57E-06
32	322734	P	32	364273	-2	1.06E-05
31	65830	F	31	324346	-2	3.98E-05
31	73321	P	31	82843	-2	3.98E-05
31	97001	P	31	291068	-2	3.98E-05
31	347965	F	31	348022	-2	3.98E-05
33	343843	R	33	343853	-3	8.77E-05
30	16746	P	30	76594	-2	1.49E-04
30	21013	F	30	238850	-2	1.49E-04

30	29062	P	30	381735	-2	1.49E-04
30	30729	F	30	114263	-2	1.49E-04
30	102072	F	30	347348	-2	1.49E-04
30	125949	P	30	125949	-2	1.49E-04
30	181041	F	30	326837	-2	1.49E-04
30	221831	F	30	258010	-2	1.49E-04
30	347910	F	30	348039	-2	1.49E-04
32	159845	P	32	364237	-3	3.19E-04
31	18585	F	31	311786	-3	1.16E-03
31	52699	P	31	362453	-3	1.16E-03
31	118121	F	31	393456	-3	1.16E-03
31	130772	P	31	145655	-3	1.16E-03
31	181072	P	31	363848	-3	1.16E-03
31	216089	F	31	311697	-3	1.16E-03
31	265986	P	31	265986	-3	1.16E-03
31	352557	F	31	356081	-3	1.16E-03
30	41223	F	30	181045	-3	4.18E-03
30	111628	P	30	243621	-3	4.18E-03
30	112413	F	30	234129	-3	4.18E-03
30	118130	F	30	321828	-3	4.18E-03
30	120189	F	30	314094	-3	4.18E-03
30	173384	F	30	340862	-3	4.18E-03
30	177701	F	30	391914	-3	4.18E-03
30	193472	F	30	311385	-3	4.18E-03
30	229623	F	30	276748	-3	4.18E-03

Table S8 Dispersed repeats in the *T. latifolia* plastome.

The repeat length of the first part	The starting site of the first part	Matching direction	The repeat length of the second part	The starting site of the second part	interval distance of repeats	E-value
26930	89131	P	26930	134615	0	0.00E+00
65	49427	F	65	49464	-1	1.05E-27
57	96894	F	57	96918	-3	2.79E-19
57	96894	P	57	153701	-3	2.79E-19
57	96918	P	57	153725	-3	2.79E-19
57	153705	F	57	153729	-3	2.79E-19
49	94468	F	49	94489	-2	2.45E-16
49	94468	P	49	156138	-2	2.45E-16
49	94489	P	49	156159	-2	2.45E-16
49	156138	F	49	156159	-2	2.45E-16
46	153716	F	46	153740	-2	1.38E-14
36	15417	P	36	15417	0	1.55E-12
39	46253	F	39	104401	-1	2.84E-12
39	46253	P	39	146236	-1	2.84E-12
38	5	P	38	5	-2	6.15E-10
38	63445	P	38	63445	-2	6.15E-10
38	63459	P	38	63459	-2	6.15E-10
38	96913	F	38	96937	-2	6.15E-10
38	96913	P	38	153701	-2	6.15E-10
38	96937	P	38	153725	-2	6.15E-10
37	96890	F	37	96938	-2	2.33E-09
37	96890	P	37	153701	-2	2.33E-09
37	96938	P	37	153749	-2	2.33E-09
37	153701	F	37	153749	-2	2.33E-09
30	9711	P	30	9711	0	6.37E-09
30	85759	F	30	85774	0	6.37E-09
30	120752	P	30	120752	0	6.37E-09
38	6148	P	38	6154	-3	2.21E-08
34	1346	P	34	1346	-2	1.26E-07
31	8396	P	31	47759	-1	1.48E-07
33	99942	F	33	99967	-2	4.73E-07
33	99942	P	33	150676	-2	4.73E-07
33	99967	P	33	150701	-2	4.73E-07
33	150676	F	33	150701	-2	4.73E-07

35	9747	F	35	68178	-3	1.10E-06
32	89182	P	32	89182	-2	1.78E-06
32	89182	F	32	161462	-2	1.78E-06
32	161462	P	32	161462	-2	1.78E-06
31	9163	P	31	63457	-2	6.66E-06
33	9182	P	33	63421	-3	1.47E-05
33	9761	P	33	9761	-3	1.47E-05
33	31387	P	33	31387	-3	1.47E-05
30	5987	P	30	5987	-2	2.49E-05
30	9168	P	30	9758	-2	2.49E-05
30	9767	F	30	63443	-2	2.49E-05
30	48848	P	30	48848	-2	2.49E-05
30	51693	F	30	51718	-2	2.49E-05
30	123834	F	30	123862	-2	2.49E-05
32	9767	P	32	123673	-3	5.33E-05
32	9869	P	32	123696	-3	5.33E-05
31	8396	F	31	38100	-3	1.93E-04
31	9122	F	31	9146	-3	1.93E-04
31	9174	F	31	9758	-3	1.93E-04
31	9769	P	31	63452	-3	1.93E-04
31	9845	F	31	9884	-3	1.93E-04
31	41314	F	31	43538	-3	1.93E-04
31	49401	P	31	63421	-3	1.93E-04
31	49424	F	31	49498	-3	1.93E-04
30	8949	F	30	8979	-3	6.98E-04
30	9163	F	30	63468	-3	6.98E-04
30	9762	P	30	63441	-3	6.98E-04
30	9815	F	30	9835	-3	6.98E-04
30	9819	P	30	87450	-3	6.98E-04
30	9861	F	30	9878	-3	6.98E-04
30	10964	F	30	39085	-3	6.98E-04
30	15359	P	30	15373	-3	6.98E-04
30	41355	F	30	43579	-3	6.98E-04
30	48837	F	30	63442	-3	6.98E-04
30	49448	P	30	63439	-3	6.98E-04
30	49485	P	30	63439	-3	6.98E-04
30	76569	F	30	76572	-3	6.98E-04
30	94490	F	30	94511	-3	6.98E-04
30	94490	P	30	156135	-3	6.98E-04
30	94511	P	30	156156	-3	6.98E-04
30	123753	F	30	123772	-3	6.98E-04

Table S9 Dispersed repeats in the *T. domingensis* mitogenome.

The repeat length of the first part	The starting site of the first part	Matching direction	The repeat length of the second part	The starting site of the second part	interval distance of repeats	E-value
686	147262	F	686	338702	0	0.00E+00
658	91181	P	658	195876	0	0.00E+00
524	188535	F	524	367824	0	0.00E+00
337	146818	F	337	338260	-3	9.56E-185
302	146853	F	302	338295	-2	2.71E-166
272	277392	F	272	356356	0	7.63E-154
240	146770	F	240	338212	-3	8.64E-127
220	48142	P	220	111134	-1	1.02E-119
199	3581	F	199	42082	0	6.80E-110
202	273505	P	202	319239	-1	6.44E-109
195	163149	P	195	368556	0	1.74E-107
199	192294	F	199	266981	-1	4.06E-107
203	9155	F	203	25678	-2	4.90E-107
191	146298	F	191	264330	0	4.46E-105
191	273516	P	191	319239	0	4.46E-105
189	192304	F	189	266991	0	7.13E-104
199	192576	F	199	267251	-3	2.38E-102
184	146668	F	184	338110	-3	2.01E-93
179	146632	F	179	338074	-3	1.90E-90
161	229750	F	161	267440	0	5.14E-87
153	192824	F	153	267591	0	3.37E-82
160	146657	F	160	338099	-3	3.72E-79
155	146614	F	155	338056	-3	3.46E-76
140	147015	F	140	338457	0	2.26E-74
141	164033	F	141	310612	-1	2.39E-72
133	242256	P	133	368780	0	3.70E-70
127	129309	F	127	196534	0	1.52E-66
130	197644	F	130	337019	-2	1.79E-63
124	135549	P	124	244779	-1	3.61E-62
116	48362	F	116	245812	-2	3.82E-55
116	97179	F	116	228116	-2	3.82E-55
95	1637	P	95	335869	0	2.80E-47
95	17847	F	95	64765	0	2.80E-47
93	135580	P	93	244779	0	4.48E-46
90	81524	P	90	193041	0	2.87E-44
89	310534	F	89	344372	0	1.15E-43

84	188990	F	84	263017	0	1.17E-40
94	146307	P	94	163842	-3	4.05E-40
94	163842	P	94	264339	-3	4.05E-40
83	238921	F	83	315023	0	4.69E-40
93	175523	P	93	369867	-3	1.57E-39
90	146315	P	90	163838	-3	9.09E-38
82	245806	P	82	264929	-1	4.62E-37
81	28946	P	81	333882	-1	1.83E-36
81	317487	P	81	370689	-1	1.83E-36
82	175534	P	82	369867	-2	5.61E-35
74	127120	F	74	204050	0	1.23E-34
81	246184	F	81	348122	-2	2.19E-34
73	191635	F	73	192077	0	4.92E-34
80	147191	F	80	338630	-2	8.55E-34
76	48291	P	76	111129	-1	1.75E-33
76	48362	P	76	264929	-1	1.75E-33
75	337763	F	75	337805	-1	6.92E-33
74	31933	F	74	233899	-1	2.73E-32
77	104276	F	77	338245	-2	5.06E-32
80	246456	F	80	348391	-3	6.67E-32
69	263017	F	69	368279	0	1.26E-31
78	246444	F	78	348379	-3	9.87E-31
71	147200	F	71	338639	-1	1.68E-30
71	169078	F	71	240390	-1	1.68E-30
77	104276	F	77	146803	-3	3.80E-30
66	192506	F	66	267193	0	8.07E-30
66	359140	F	66	373449	0	8.07E-30
64	164110	F	64	310689	0	1.29E-28
67	48487	F	67	245937	-1	4.05E-28
63	169086	F	63	240398	0	5.16E-28
62	358123	F	62	358704	0	2.06E-27
69	25185	P	69	124125	-2	2.66E-27
61	264583	P	61	389758	0	8.26E-27
68	104285	F	68	146812	-2	1.03E-26
71	36885	P	71	169706	-3	1.22E-26
71	146585	F	71	338027	-3	1.22E-26
59	72853	P	59	209935	0	1.32E-25
62	42521	P	62	119026	-1	3.84E-25
58	43148	F	58	188909	0	5.29E-25
58	43148	F	58	368198	0	5.29E-25
67	197392	F	67	337333	-3	2.61E-24
56	9118	P	56	190910	0	8.46E-24
65	129189	P	65	163341	-3	3.80E-23
64	146603	F	64	338045	-3	1.45E-22

53	31954	F	53	233920	0	5.41E-22
53	243612	F	53	297531	0	5.41E-22
63	197407	F	63	337348	-3	5.53E-22
56	129198	P	56	163341	-1	1.42E-21
59	175619	P	59	369805	-2	2.03E-21
54	25608	P	54	299156	-1	2.19E-20
53	135509	P	53	246532	-1	8.61E-20
59	175631	P	59	369793	-3	1.16E-19
49	26842	P	49	349928	0	1.39E-19
46	48392	P	46	264929	0	8.87E-18
46	54411	P	46	62196	0	8.87E-18
46	245842	P	46	264929	0	8.87E-18
49	25205	P	49	124125	-1	2.04E-17
48	36908	P	48	169706	-1	7.98E-17
48	146543	F	48	337980	-1	7.98E-17
44	147152	F	44	258059	0	1.42E-16
50	246492	F	50	348427	-2	3.82E-16
43	42540	P	43	119026	0	5.68E-16
51	175579	P	51	369853	-3	4.87E-15
51	189195	P	51	280063	-3	4.87E-15
41	317527	P	41	370689	0	9.08E-15
47	188921	P	47	347435	-2	2.16E-14
47	347435	P	47	368210	-2	2.16E-14
40	25214	P	40	124125	0	3.63E-14
40	308489	F	40	337185	0	3.63E-14
43	146611	F	43	266415	-1	7.32E-14
46	43160	P	46	347436	-2	8.26E-14
39	86919	F	39	211906	0	1.45E-13
38	147233	F	38	338672	0	5.81E-13
47	211967	F	47	333642	-3	9.71E-13
41	48993	P	41	171501	-1	1.12E-12
41	189205	P	41	280063	-1	1.12E-12
44	46031	P	44	263136	-2	1.21E-12
37	146505	F	37	264530	0	2.32E-12
46	246287	F	46	348216	-3	3.63E-12
40	121020	P	40	313163	-1	4.36E-12
36	197742	P	36	295573	0	9.30E-12
36	247365	P	36	317429	0	9.30E-12
35	63699	F	35	86930	0	3.72E-11
44	189186	P	44	280079	-3	5.07E-11
38	223492	F	38	333014	-1	6.63E-11
41	35097	F	41	383112	-2	6.70E-11
41	104799	P	41	242638	-2	6.70E-11
34	42045	P	34	42045	0	1.49E-10

34	82735	P	34	91301	0	1.49E-10
34	82735	F	34	196380	0	1.49E-10
34	104319	F	34	146846	0	1.49E-10
34	107993	P	34	257184	0	1.49E-10
40	12035	P	40	12035	-2	2.55E-10
40	266418	F	40	338056	-2	2.55E-10
37	197472	F	37	337413	-1	2.58E-10
42	48954	F	42	183313	-3	7.04E-10
42	264529	F	42	337942	-3	7.04E-10
39	136439	P	39	387907	-2	9.69E-10
32	129222	P	32	163341	0	2.38E-09
38	91336	F	38	318996	-2	3.68E-09
38	146505	F	38	337943	-2	3.68E-09
38	196341	P	38	318996	-2	3.68E-09
35	6028	F	35	334630	-1	3.91E-09
31	94397	F	31	186299	0	9.52E-09
31	146623	F	31	266427	0	9.52E-09
31	230236	P	31	247500	0	9.52E-09
40	11692	P	40	51554	-3	9.69E-09
37	175653	P	37	369793	-2	1.39E-08
34	104319	F	34	338288	-1	1.52E-08
30	1930	P	30	1930	0	3.81E-08
30	3701	P	30	8346	0	3.81E-08
30	8346	P	30	42202	0	3.81E-08
30	101974	F	30	317451	0	3.81E-08
33	48445	F	33	245895	-1	5.89E-08
33	84865	F	33	287709	-1	5.89E-08
33	337763	F	33	337847	-1	5.89E-08
35	85287	F	35	304396	-2	1.99E-07
35	246298	F	35	348227	-2	1.99E-07
32	94741	F	32	186624	-1	2.29E-07
32	188936	P	32	347435	-1	2.29E-07
37	167713	F	37	242356	-3	4.88E-07
31	43175	P	31	347436	-1	8.86E-07
31	266427	F	31	338065	-1	8.86E-07
36	242433	P	36	297403	-3	1.79E-06
33	42519	F	33	133938	-2	2.83E-06
30	63697	F	30	211915	-1	3.43E-06
30	144711	F	30	145066	-1	3.43E-06
35	62616	F	35	273092	-3	6.57E-06
32	295577	P	32	337117	-2	1.06E-05
31	167711	P	31	368784	-2	3.99E-05
31	181869	P	31	191391	-2	3.99E-05
31	198882	F	31	335506	-2	3.99E-05

31	311829	F	31	311886	-2	3.99E-05
33	315996	R	33	316006	-3	8.77E-05
30	6703	F	30	42883	-2	1.49E-04
30	25863	F	30	243701	-2	1.49E-04
30	83673	F	30	333016	-2	1.49E-04
30	138764	P	30	138764	-2	1.49E-04
30	150451	F	30	233985	-2	1.49E-04
30	162641	F	30	312504	-2	1.49E-04
30	188119	P	30	247968	-2	1.49E-04
30	235653	P	30	278115	-2	1.49E-04
30	311813	F	30	311942	-2	1.49E-04
32	104867	P	32	295613	-3	3.19E-04
31	48624	F	31	348155	-3	1.16E-03
31	48968	F	31	183327	-3	1.16E-03
31	83641	P	31	296003	-3	1.16E-03
31	119057	P	31	133940	-3	1.16E-03
31	146591	F	31	266395	-3	1.16E-03
31	212013	P	31	297398	-3	1.16E-03
31	246128	F	31	348066	-3	1.16E-03
31	303776	F	31	307300	-3	1.16E-03
31	393866	P	31	393866	-3	1.16E-03
30	21092	P	30	153085	-3	4.18E-03
30	30584	F	30	152300	-3	4.18E-03
30	42527	F	30	133946	-3	4.18E-03
30	71242	F	30	348468	-3	4.18E-03
30	83673	F	30	223494	-3	4.18E-03
30	87013	F	30	267938	-3	4.18E-03
30	121034	P	30	313159	-3	4.18E-03
30	144524	F	30	345759	-3	4.18E-03
30	196355	P	30	318990	-3	4.18E-03

Table S10 Dispersed repeats in the *T. domingensis* plastome.

The repeat length of the first part	The starting site of the first part	Matching direction	The repeat length of the second part	The starting site of the second part	interval distance of repeats	E-value
26882	0	P	26882	45384	0	0.00E+00
57	7740	F	57	7764	-3	2.78E-19

57	7740	P	57	64445	-3	2.78E-19
57	7764	P	57	64469	-3	2.78E-19
57	64449	F	57	64473	-3	2.78E-19
46	111793	F	46	111834	0	1.48E-18
40	151367	F	40	151387	0	6.05E-15
46	64460	F	46	64484	-2	1.38E-14
36	145801	P	36	145801	0	1.55E-12
39	15242	P	39	115015	-1	2.83E-12
39	56985	F	39	115015	-1	2.83E-12
38	7759	F	38	7783	-2	6.12E-10
38	7759	P	38	64445	-2	6.12E-10
38	7783	P	38	64469	-2	6.12E-10
38	97834	P	38	97834	-2	6.12E-10
38	97848	P	38	97848	-2	6.12E-10
38	161182	P	38	161182	-2	6.12E-10
37	7736	F	37	7784	-2	2.32E-09
37	7736	P	37	64445	-2	2.32E-09
37	7784	P	37	64493	-2	2.32E-09
37	64445	F	37	64493	-2	2.32E-09
30	40676	P	30	40676	0	6.34E-09
30	151504	P	30	151504	0	6.34E-09
38	155046	P	38	155052	-3	2.20E-08
34	145759	F	34	145778	-2	1.25E-07
34	159845	P	34	159845	-2	1.25E-07
31	75587	F	31	75601	-1	1.47E-07
31	113517	P	31	152817	-1	1.47E-07
33	10785	F	33	10810	-2	4.71E-07
33	10785	P	33	61423	-2	4.71E-07
33	10810	P	33	61448	-2	4.71E-07
33	61423	F	33	61448	-2	4.71E-07
35	93134	F	35	151463	-3	1.09E-06
32	43	P	32	43	-2	1.77E-06
32	43	F	32	72191	-2	1.77E-06
32	72191	P	32	72191	-2	1.77E-06
31	97843	P	31	152050	-2	6.63E-06
33	97877	P	33	152029	-3	1.46E-05
33	129844	P	33	129844	-3	1.46E-05
30	37549	F	30	37577	-2	2.48E-05
30	109561	F	30	109586	-2	2.48E-05
30	111774	F	30	111811	-2	2.48E-05
30	112429	P	30	112429	-2	2.48E-05
30	151333	F	30	151355	-2	2.48E-05
30	151457	P	30	152046	-2	2.48E-05
30	155221	P	30	155221	-2	2.48E-05

32	151455	F	32	152038	-3	5.31E-05
31	97879	P	31	111875	-3	1.92E-04
31	111774	F	31	111852	-3	1.92E-04
31	117730	F	31	119951	-3	1.92E-04
31	123157	F	31	152817	-3	1.92E-04
31	152067	F	31	152091	-3	1.92E-04
30	37639	F	30	37658	-3	6.95E-04
30	73917	P	30	151400	-3	6.95E-04
30	97834	F	30	152052	-3	6.95E-04
30	97859	F	30	112440	-3	6.95E-04
30	97862	P	30	111788	-3	6.95E-04
30	117690	F	30	119911	-3	6.95E-04
30	122175	F	30	150258	-3	6.95E-04
30	145851	P	30	145865	-3	6.95E-04
30	151364	F	30	151404	-3	6.95E-04
30	151447	P	30	157873	-3	6.95E-04
30	152236	F	30	152266	-3	6.95E-04

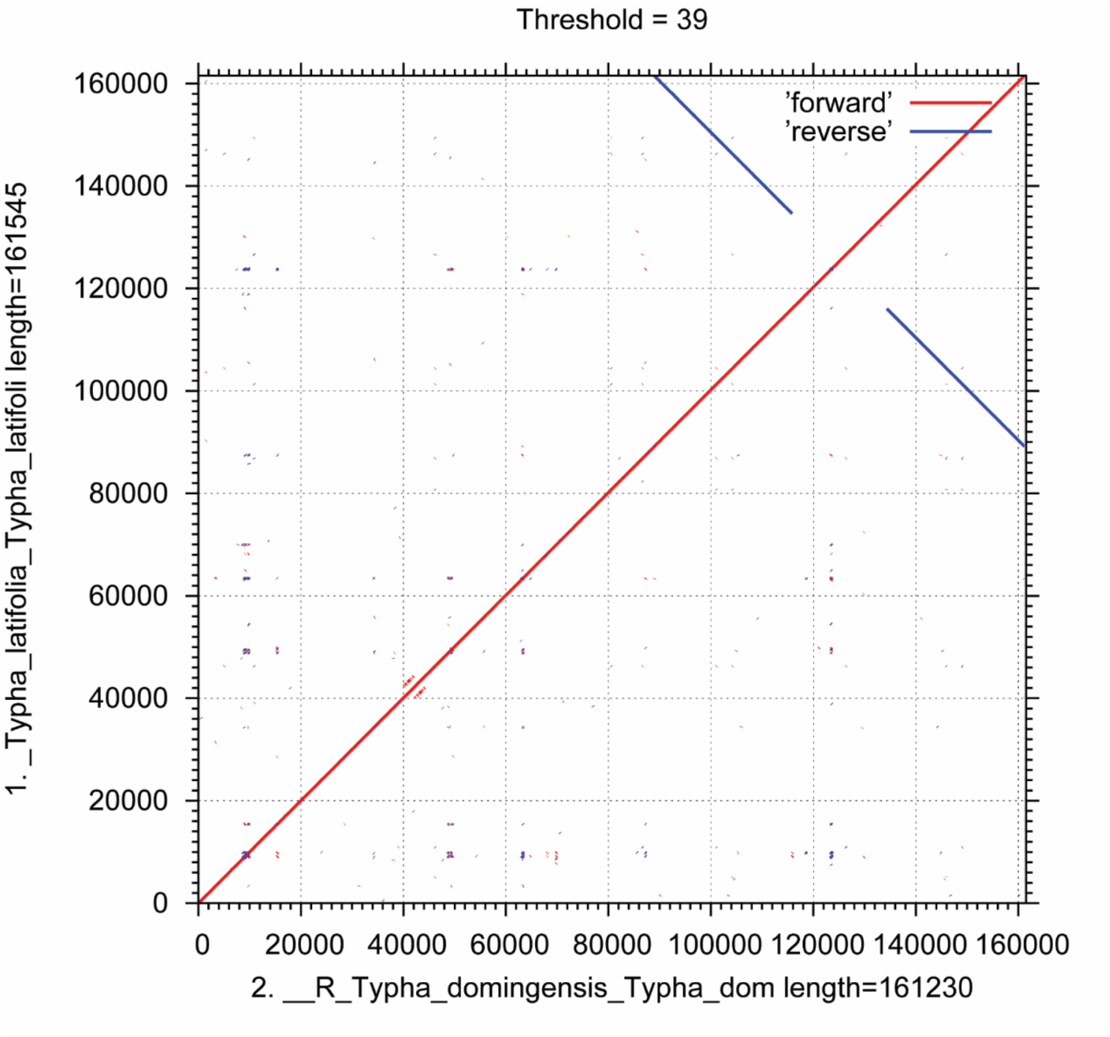

Supplementary Figure 1. The dot plot of two *Typha* plastomes. The horizontal line represents the plastome assembled in this study *Typha latifolia* and the vertical line represents the one *Typha domingensis*.

Table S11 DNA transfer of *Typha latifolia* organelle genomes.

Sequence similarity between the plastome and the mitogenome were analyzed to identify transferred DNA fragments by using BLASTN with an e-value cut-off of 1e-5.

Group	length	Chloroplast genome		mitogenome		E-value	Chloroplast coding region	Chloroplast coding length	Chloroplast coding genes	Mitogenome coding region	Mitogenome coding length	Mitogenome coding genes	Direction (mitogenome)
		Start	End	Start	End								
I	949	86496	87427	143247	142324	0	*rpl16*	1492	*rpl16*	Null	0	Null	Null
II	437	95158	95594	205244	204816	0	*ycfII*	6882	*ycfII*	Null	0	Null	Null
	437	155083	155519	204816	205244	0	*ycfII*	6882	*ycfII*	Null	0	Null	Null
III	436	85814	86248	84063	84490	8.54E-140	*rpl14*	369	*rpl14*	Null	0	Null	Null
IV	460	71255	71692	205336	205790	1.96E-96	*trnW-CCA* *trnP-UGG*	74	*trnW-CCA* *trnP-UGG*	*trnW-CCA* *trnP-UGG*	74 75	*trnW-CCA*, *trnP-UGG*	Negative
V	288	36020	36307	293093	293362	5.80E-52	*psbD*	1062	*psbD*	Null	0	Null	Null
VI	135	85500	85633	83838	83972	4.58E-38	*rps8*	405	*rps8*	*trnN-GUU*	72	*trnN-GUU*	Negative
VII	111	76114	76224	221349	221242	7.71E-31	*clpP1*	2107	*clpP1*	Null	0	Null	Null
VIII	93	136003	136095	35734	35642	1.27E-38	*trnN-GUU*	72	*rps8*	Null	0	Null	Null
	93	114582	114674	35642	35734	1.27E-38	Null	0	Null	*trnH-GUG*	74	*trnH-GUG*	Negative
IX	97	110740	110836	363707	363611	7.82E-21	Null	0	Null	*rrn26*	3414	*rrn26*	Negative

	97	139841	139937	363611	363707	7.82E-21	*rrn23*	28112	*rrn23*	*rrn26*	3414	*rrn26*	Negative
X	91	87470	87557	142331	142245	6.13E-12	*rpl16*	1492	*rpl16*	Null	0	Null	Null
XI	96	124001	124094	112823	112913	2.21E-11	*ndhE*	306	*ndhE*	Null	0	Null	Null
XII	83	160928	161010	261461	261543	4.61E-33	*trnH-GUG*	75	*trnH-GUG*	*trnH-GUG*	74	*trnH-GUG*	Positive
	83	89667	89749	261461	261543	4.61E-33	*trnH-GUG*	75	*trnH-GUG*	*trnH-GUG*	74	*trnH-GUG*	Positive

Table S12 DNA transfer of *Typha domingensis* organelle genomes.

Sequence similarity between the plastome and the mitogenome were analyzed to identify transferred DNA fragments by using BLASTN with an e-value cut-off of 1e-5.

Group	length	Chloroplast genome		mitogenome		E-value	Chloroplast coding region	Chloroplast coding length	Chloroplast coding genes	Mitogenome coding region	Mitogenome coding length	Mitogenome coding genes	Direction (mitogenome)
		Start	End	Start	End								
I	976	73949	74897	122448	121497	0	*rps12*	913	*rps12*	Null	0	Null	Null
II	437	6005	6441	59501	59929	0	Null	0	Null	Null	0	Null	Null
	437	65826	66262	59929	59501	0	*cemA*	477	*cemA*	Null	0	Null	Null
III	436	75144	75578	180254	180681	8.52E-140	*clpP1*	2093	*clpP1*	Null	0	Null	Null
IV	464	89662	90102	58952	59409	1.95E-96	*rpl2*	1506	*rpl2*	*trnW-CCA* *trnP-UGG*	74 75	*trnW-CCA*, *trnP-UGG*	Positive

V	285	124977	125261	366522	366788	5.78E-52	*ndhI*	543	*ndhI*	Null	0	Null	Null
VI	135	75758	75891	180772	180906	4.57E-38	*clpP1*	2093	*clpP1*	Null	0	Null	Negative
VII	110	85149	85258	43503	43396	1.65E-32	Null	0	Null	Null	0	Null	Null
VIII	93	46771	46863	229010	229102	1.27E-38	*ycfI*	1972	*ycfI*	*trnN- GUU*	72	*trnN- GUU*	Positive
	93	25404	25496	229102	229010	1.27E-38	Null	0	Null	*trnN- GUU*	72	*trnN- GUU*	Positive
IX	97	21565	21661	296176	296272	7.81E-21	Null	0	Null	Null	0	Null	Null
	97	50606	50702	296272	296176	7.81E-21	*trnL-UAA*	643	*trnL-UAA*	Null	0	Null	Null
X	91	73841	73928	122499	122413	6.12E-12	*rpl20*	354	*rpl20*	Null	0	Null	Null
XI	96	37356	37449	151831	151921	2.20E-11	*psbC*	951	*psbC*	Null	0	Null	Null
XII	83	71660	71742	3201	3283	4.60E-33	Null	0	Null	*trnH-GUG*	74	*trnH-GUG*	Negative
	83	525	607	3283	3201	4.60E-33	*psbA*	1062	*psbA*	*trnH-GUG*	74	*trnH-GUG*	Negative
